# Nonlocal and local models for taxis in cell migration: a rigorous limit procedure

**DOI:** 10.1007/s00285-020-01536-4

**Published:** 2020-10-17

**Authors:** Maria Eckardt, Kevin J. Painter, Christina Surulescu, Anna Zhigun

**Affiliations:** 1 Felix-Klein-Zentrum für Mathematik, Technische Universität Kaiserslautern, Paul-Ehrlich-Str. 31, 67663 Kaiserslautern, Germany; 2 Department of Mathematics & Maxwell Institute, Heriot-Watt University, Edinburgh, EH14 4AS Scotland, UK; 3 School of Mathematics and Physics, Queen’s University Belfast, University Road, Belfast, BT7 1NN Northern Ireland, UK

**Keywords:** Cell–cell and cell–tissue adhesion, Nonlocal and local chemotaxis, Haptotaxis, Integro-differential equations, Unified approach, Global existence, Rigorous limit behaviour, Weak solutions, 35Q92, 92C17, 35K55, 35R09, 47G20, 35B45, 35D30

## Abstract

A rigorous limit procedure is presented which links nonlocal models involving adhesion or nonlocal chemotaxis to their local counterparts featuring haptotaxis and classical chemotaxis, respectively. It relies on a novel reformulation of the involved nonlocalities in terms of integral operators applied directly to the gradients of signal-dependent quantities. The proposed approach handles both model types in a unified way and extends the previous mathematical framework to settings that allow for general solution-dependent coefficient functions. The previous forms of nonlocal operators are compared with the new ones introduced in this paper and the advantages of the latter are highlighted by concrete examples. Numerical simulations in 1D provide an illustration of some of the theoretical findings.

## Introduction

Macroscopic equations and systems describing the evolution of populations in response to soluble and insoluble environmental cues have been intensively studied and the palette of such reaction-diffusion-taxis models is continuously expanding. Models of such form are motivated by problems arising in various contexts, a large part related to cell migration and proliferation connected to tumor invasion, embryonal development, wound healing, biofilm formation, insect behavior in response to chemical cues, etc. We refer, e.g. to Bellomo et al. (2015) for a recent review also containing some deduction methods for taxis equations based on kinetic transport equations.

Apart from such purely local PDE systems with taxis, several spatially nonlocal models have been introduced over the last two decades and are attracting ever increasing interest. They involve integro-differential operators in one or several terms of the featured reaction-diffusion-drift equations. Their aim is to characterize interactions between individuals or signal perception happening not only at a specific location, but over a whole set (usually a ball) containing (centered at) that location. In the context of cell populations, for instance, this seems to be a more realistic modeling assumption, as cells are able to extend various protrusions (such as lamellipodia, filopodia, cytonemes, etc.) into their surroundings, which can reach across long distances compared against cell size, see González-Méndez et al. (2017) and Sáenz-de Santa-María et al. (2017) and references therein. Moreover, the cells are able to relay signals they perceive and thus transmit them to cells with which they are not in direct contact, thereby influencing their motility, see e.g., Eom and Parichy (2017) and Garcia and Parent (2008). Cell–cell and cell–tissue adhesion are essential for mutual communication, homeostasis, migration, proliferation, sorting, and many other biological processes. A large variety of models for adhesive behavior at the cellular level have been developed to account for the dynamics of focal contacts, e.g. Bell (1978), Bell et al. (1984) and Ward and Hammer (1993) and to assess their influence on cytoskeleton restructuring and cell migration, e.g. DiMilla et al. (1991), Dickinson and Tranquillo (1993), Kuusela and Alt (2008), Uatay (2019). Continuous, spatially nonlocal models involving adhesion were introduced more recently (Armstrong et al. 2006) and are attracting increasing interest from the modeling (Bitsouni et al. 2018; Buttenschön et al. 2018; Carrillo et al. 2019; Domschke et al. 2014; Gerisch and Chaplain 2008; Gerisch and Painter 2010; Murakawa and Togashi 2015; Painter et al. 2010), analytical (Chaplain et al. 2011; Dyson et al. 2013, 2010; Sherratt et al. 2009; Hillen et al. 2018), and numerical (Gerisch 2010) viewpoints. Yet more recent models (Domschke et al. 2017; Engwer et al. 2017) also take into account subcellular level dynamics, thus involving further nonlocalities (besides adhesion), with respect to some structure variable referring to individual cell state. Thereby, multiscale mathematical settings are obtained, which lead to challenging problems for analysis and numerics. Another essential aspect of cell migration is the directional bias in response to a diffusing signal, commonly termed chemotaxis. A model of cell migration with finite sensing radius, thus featuring nonlocal chemotaxis has been introduced in Othmer and Hillen (2002) and readdressed in Hillen et al. (2007) from the perspective of well-posedness, long time behaviour, and patterning. We also refer to Loy and Preziosi (2019) for further spatially nonlocal models and their formal deduction.

For adhesion and nonlocal chemotaxis models, a gradient of some nondiffusing or diffusing signal is replaced by a nonlocal integral term. Here we are only interested in this type of model, and refer to Chen et al. (2020); Eftimie (2018), Kavallaris and Suzuki (2018) for reviews on settings involving other types of nonlocality. Specifically, following Armstrong et al. (2006), Gerisch and Chaplain (2008), Hillen et al. (2007) and Othmer and Hillen (2002), we consider the subsequent systems, whose precise mathematical formulations will be specified further below:

1. a prototypical nonlocal model for adhesion
  1.1a $$\begin{aligned} \partial _t c_{r}&=\nabla \cdot \left( D_c(c_{r},v_{r})\nabla c_{r}-c_{r}\chi (c_{r},v_{r}) \mathcal{A}_{r}(g(c_r,v_r))\right) +f_c(c_{r},v_{r}), \end{aligned}$$
  1.1b $$\begin{aligned} \partial _t v_{r}&=f_v(c_{r},v_{r}), \end{aligned}$$
  where 1.2 is referred to as the adhesion velocity, and the function $F_r$ describes how the magnitude of the interaction force depends on the interaction range $|\xi |$ within the sensing radius *r*. We require this function to satisfy
  **Assumptions 1.1** (Assumptions on $F_r$)
  (i) $(r,\rho )\mapsto F_r(\rho )$ *is continuous and positive in* $\left[ 0,r_0\right] ^2$ *for some* $r_0>0$;
  (ii) $$F_0(0)={ n+1}$$.^1^
  The quantity
  $$\begin{aligned} {\mathbb {F}}(c_r,v_r)=c_r\chi (c_r,v_r){{{\mathcal {A}}}}_{r}(g(c_r,v_r)) \end{aligned}$$
  is often referred to as the total adhesion flux, possibly scaled by some constant involving the typical cell size or the sensing radius, see e.g., Armstrong et al. (2006) and Buttenschön et al. (2018). Here we also include a coefficient $\chi (c_r,v_r)$ that depends on cell and tissue (extracellular matrix, ECM) densities, which can be seen as characterizing the sensitivity of cells towards their neighbours and the surrounding tissue. It will, moreover, help provide in a rather general framework a unified presentation of this and the subsequent local and nonlocal model classes for adhesion, haptotactic, and chemotactic behavior of moving cells. System (1.1) is a simplification of the integro-differential system (4) in Gerisch and Chaplain (2008). The main difference between the two settings is that in our case we ignore the so-called matrix-degrading enzymes (MDEs). Instead, we assume the cells to degrade the tissue directly: this fairly standard simplification (e.g., Painter et al. 2010) effectively assumes that proteolytic enzymes remain localised to the cells, and helps simplify the analysis. On the other hand, (1.1) can also be viewed as a nonlocal version of the haptotaxis model with nonlinear diffusion:
  1.3a $$\begin{aligned}&\partial _t c=\nabla \cdot \left( D_c(c,v)\nabla c-c\chi (c,v) \nabla g(c,v)\right) +f_c(c,v), \end{aligned}$$
  1.3b $$\begin{aligned}&\partial _t v=f_v(c,v); \end{aligned}$$
  2. a prototypical nonlocal chemotaxis-growth model
  1.4a $$\begin{aligned} \partial _t c_r&=\nabla \cdot \left( D_c(c_{r},v_{r})\nabla c_{r}-c_{r}\chi (c_{r},v_{r}) \mathring{\nabla }_{ r}v_{r}\right) +f_c(c_{r},v_{r}), \end{aligned}$$
  1.4b $$\begin{aligned} \partial _t v_r&=D_v\varDelta v_r+f_v(c_{r},v_{r}) \end{aligned}$$
  with the nonlocal gradient  System (1.4) can be seen as a nonlocal version of the chemotaxis-growth model
  1.5a $$\begin{aligned} \partial _t c&=\nabla \cdot \left( D_c(c,v)\nabla c-c\chi (c,v) \nabla v\right) +f_c(c,v), \end{aligned}$$
  1.5b $$\begin{aligned} \partial _t v&=D_v\varDelta v+f_v(c,v), \end{aligned}$$
  where $\chi (c,v)$ is the chemotactic sensitivity function. As mentioned above, in order to have a unified description of our systems (1.3) and (1.5) and of their respective nonlocal counterparts (1.1) and (1.4), we later introduce a more general version of the nonlocal chemotaxis flux, similar to the above adhesion velocity ${\mathcal {A}}_r$.

Here and below $B_r$ and $S_r$ denote the open *r*-ball and the *r*-sphere in $\mathbb {R}^n$, both centred at the origin, andare the usual mean values of a function *u* over $B_r$ and $S_r$, respectively. The nonlocal systems (1.3) and (1.5) are stated for

$$\begin{aligned} t>0,\quad x\in \varOmega \subset \mathbb {R}^n. \end{aligned}$$

Unless the spatial domain $\varOmega$ is the whole $\mathbb {R}^n$, suitable boundary conditions are required. In the latter case, usually periodicity is assumed, which is not biologically realistic in general. Still, this offers the easiest way to properly define the output of the nonlocal operator in the boundary layer where the sensing region is not fully contained in $\varOmega$. Very recently various other boundary conditions have been derived and compared in the context of a single equation modeling cell–cell adhesion in 1D (Hillen and Buttenschön 2020).

Few previous works focus on solvability for models with nonlocality in a taxis term. Some of them deal with single equations that only involve cell–cell adhesion (Dyson et al. 2010, 2013; Hillen and Buttenschön 2020), others study nonlocal systems of the sort considered here for two (Hillen et al. 2007) or more components (Engwer et al. 2017). The global solvability and boundedness study in Hillen et al. (2018) is obtained for the case of a nonlocal operator with integration over a set of sampling directions being an open, not necessarily strict subset of $\mathbb {R}^{n}$. The systems studied there include settings with a third equation for the dynamics of diffusing MDEs. Conditions which secure uniform boundedness of solutions to such cell–cell and cell–tissue adhesion models in 1D were elaborated in Sherratt et al. (2009).

Some heuristic analysis via local Taylor expansions was performed in Gerisch and Chaplain (2008) and Hillen (2007) showing that as $r\rightarrow 0$ the outputs ${{{\mathcal {A}}}}_{r}u$ and $\mathring{\nabla }_{r}u$, respectively, converge pointwise to $\nabla u$ for a fixed and sufficiently smooth *u*. In Hillen et al. (2007) it was observed that it would be interesting to study rigorously the limiting behaviour of solutions of the nonlocal problems involving $\mathring{\nabla }_{r}u$. The authors ask in which sense, if at all, do these solutions converge to solutions of the corresponding local problem as $r\rightarrow 0$. Numerical results appeared to confirm that, in certain cases, the answer is positive. Still, to the best of our knowledge, no rigorous analytical study of this issue has as yet been performed. Clearly, any approach based on representations using Taylor polynomials requires a rather high order regularity of solution components and a suitable control on the approximation errors, and that uniformly in *r*. This is difficult or even impossible to obtain in most cases, particularly when dealing with weak solutions. In this work we propose a different approach based on the representation of the input *u* in terms of an integral of $\nabla u$ over line segments. This leads to a new description of the nonlocal operators ${{{\mathcal {A}}}}_{r}$ and $\mathring{\nabla }_{r}$ in terms of nonlocal operators applied to gradients (see Sect. 3 below). Moreover, it turns out that redefining their outputs inside the vanishing boundary layer in a suitable way allows one to perform a rigorous proof of convergence: Under suitable assumptions on the system coefficients and other parameters, appropriately defined sequences of solutions to nonlocal problems involving the mentioned modified nonlocal operators converge for $r\rightarrow 0$ to those of the corresponding local models (1.3) and (1.5), respectively. Our convergence proof is based on estimates on $c_r$ and $v_r$ which are uniform in *r* and on a compactness argument. The two models (1.1) and (1.4) are chosen as illustrations, however our idea can be further applied to other integro-differential systems with similar properties.

The rest of the paper is organised as follows. Section 2 introduces some basic notations to be used throughout this paper. In Sect. 3 we introduce the aforementioned adaptations of the nonlocal operators ${{{\mathcal {A}}}}_r$ and $\mathring{\nabla }_{r}$ and study their limiting properties as *r* becomes infinitesimally small. This turns out to be useful for our convergence proof later. We also establish in Sect. 4 the well-posedness for a certain class of equations including such operators. In the subsequent Sect. 5 we introduce a couple of nonlocal models that involve the previously considered averaging operators, prove the global existence of solutions of the respective systems, and investigate their limit behaviour as $r\rightarrow 0$. Section 6 provides some numerical simulations comparing various nonlocal and local models considered in this work in the 1D case. Finally, Sect. 7 contains a discussion of the results and a short outlook on open issues.

## Basic notations and function spaces

We denote the Lebesgue measure of a set *A* by |*A*|. Let $\varOmega \subset \mathbb {R}^n$ be a bounded domain with smooth enough boundary.

For a function $w:\varOmega \rightarrow \mathbb {R}^n$ we assume, by convention, that

$$\begin{aligned} w:=0\quad \text {in }\mathbb {R}^n\backslash {\overline{\varOmega }}. \end{aligned}$$

For $r>0$ we introduce the following subdomain of $\varOmega$

$$\begin{aligned} \varOmega _r:=\{x\in \varOmega \ :\ {\text {dist}}(x,\partial \varOmega )>r\}. \end{aligned}$$

Partial derivatives, in both classical and distributional sense, with respect to variables *t* and $x_i$, will be denoted respectively by $\partial _t$ and $\partial _{x_i}$. Further, $\nabla$, $\nabla \cdot$ and $\varDelta$ stand for the spatial gradient, divergence and Laplace operators, respectively. $\partial _{\nu }$ is the derivative with respect to the outward unit normal of $\partial \varOmega$.

We assume the reader to be familiar with the definitions and the usual properties of such spaces as: the standard Lebesgue and Sobolev spaces, spaces of functions with values in these spaces, and with anisotropic Sobolev spaces. In particular, we denote by $C_w([0,T];L^2(\varOmega ))$ the space of functions $u:[0,T]\rightarrow L^2(\varOmega )$ which are continuous w.r.t. the weak topology of $L^2(\varOmega )$.

Throughout the paper $\langle \cdot ,\cdot \rangle _{{ X^*,X}}$ denotes a duality paring between a space *X* and its dual $X^*$.

Finally, we make the following useful convention: For all indices *i*, the quantity $C_i$ denotes a positive constant or, alternatively, a positive function of its arguments. Moreover, unless explicitly stated, these constants **do not** depend upon *r*.

## Operators ${{{\mathcal {A}}}}_r$ and $\mathring{\nabla }_{r}$ and averages of $\nabla$

In this section we study the applications of the non-local operators ${{{\mathcal {A}}}}_r$ and $\mathring{\nabla }_{r}$ to fixed, i.e. independent of *r*, functions *u*. Our focus is on the limiting behaviour as $r\rightarrow 0$. Formal Taylor expansions performed in Gerisch and Chaplain (2008), Hillen et al. (2007) anticipate that the limit is the gradient operator in both cases. This we prove here rigorously under rather mild regularity assumptions on *u*. To be more precise, we replace ${{{\mathcal {A}}}}_r$ and $\mathring{\nabla }_{r}$ by certain integral operators ${{{\mathcal {T}}}}_r$ and ${{{\mathcal {S}}}}_r$ (see (3.2) and (3.7) below) applied to $\nabla u$ and show that these operators are pointwise approximations of the identity operator in the $L^p$ spaces.

We start with the operator ${{{\mathcal {A}}}}_r$. For $r\in (0,r_0]$, $u\in C^1(\varOmega )$, and $x\in \varOmega _r$ we compute that3.1Formula (3.1) extends to arbitrary $u\in W^{1,1}(\varOmega )$ by means of a density argument. Motivated by (3.1) we introduce the averaging operator3.2In Sect. 3.1 we check that ${{{\mathcal {T}}}}_rw(x)$ is well-defined for all $w\in (L^1(\varOmega ))^n$ and a.a. $x\in \varOmega$. In this notation, for all $r\in (0,r_0]$ and $u\in W^{1,1}(\varOmega )$ identity (3.1) takes the form

$$\begin{aligned} {{{\mathcal {A}}}}_ru={{{\mathcal {T}}}}_r (\nabla u)\quad \text {a.e. in }\varOmega _r. \end{aligned}$$

In the limiting case $r=0$ we have that3.3In the final step we used Assumptions 1.1(ii) which says that $F_0(0)={ n+1}$ (this explains our choice) and the trivial identity3.4Thus, we have just proved the following lemma:

### Lemma 3.1

(Adhesion velocity vs. ${{{\mathcal {T}}}}_r$) Let $u\in W^{1,1}(\varOmega )$. Then it holds that

3.5 $$\begin{aligned} {{{\mathcal {A}}}}_ru={{{\mathcal {T}}}}_r (\nabla u)\quad \text {a.e. in }\varOmega _r\quad \text {for }r\in (0,r_0]. \end{aligned}$$

Moreover, if $F_0(0)=n+1$, then

3.6 $$\begin{aligned} \nabla u={{{\mathcal {T}}}}_0 (\nabla u)\quad \text {in }\varOmega . \end{aligned}$$

In a very similar manner one can establish a representation for $\mathring{\nabla }_{r}$. For this purpose we define the averaging operator3.7The corresponding result then reads:

### Lemma 3.2

(Non-local gradient vs. ${{{\mathcal {S}}}}_r$) Let $u\in W^{1,1}(\varOmega )$. Then it holds that

3.8 $$\begin{aligned} \mathring{\nabla }_{r} u&={{{\mathcal {S}}}}_r (\nabla u)\quad \text {a.e. in }\varOmega _r\quad \text {for }r\in (0,r_0], \end{aligned}$$

3.9 $$\begin{aligned} \nabla u&={{{\mathcal {S}}}}_0 (\nabla u)\quad \text {a.e. in }\varOmega . \end{aligned}$$

The proof of Lemma 3.2 is very similar to that of Lemma 3.1 and we omit it here.

Next, we observe that identity (3.5) was established for $\varOmega _r$. In the boundary layer $\varOmega \backslash \varOmega _{r}$ the definition (1.2) of the adhesion velocity allows various extensions. For example, one could keep (1.2) by assuming (as done, e.g., in Engwer et al. (2017)) that $u:=0$ in $\mathbb {R}^n\backslash \varOmega$. An alternative would be to average over the part of the *r*-ball that lies inside the domain. Let us have a closer look at the first option (the second can be handled similarly). Consider the following example:

### Example 3.3

(${{{\mathcal {A}}}}_r$ vs. ${{{\mathcal {T}}}}_r(\nabla \cdot )$ in 1D) Let $\varOmega =(-1,1)$, $r_0=1$, $F_r\equiv 2$, and $u\equiv 1$. In this case, $u'\equiv 0$, hence

$$\begin{aligned} {{{\mathcal {T}}}}_{r}(u')\equiv 0 \equiv u'. \end{aligned}$$

For ${{{\mathcal {A}}}}_r$ one readily computes by assuming $u=0$ in $\mathbb {R}\backslash (-1,1)$ that for $x\in (-1,1)$

$$\begin{aligned} {{{\mathcal {A}}}}_ru(x)=&\frac{2}{r}\frac{1}{2r}\int _{(-1-x,1-x)\cap (-r,r)}{\text {sign}}(\xi )\,d\xi \\ =&{\left\{ \begin{array}{ll} \frac{1}{r^2}(-1+r-x)&{}\text { in }[-1,-1+r],\\ 0&{}\text { in }(-1+r,1-r)=\varOmega _r,\\ \frac{1}{r^2}(1-r-x)&{}\text { in }[1-r, 1], \end{array}\right. } \end{aligned}$$

so that

$$\begin{aligned} \Vert {{{\mathcal {A}}}}_ru\Vert _{L^1(-1,1)}&= \Vert {{{\mathcal {A}}}}_ru\Vert _{L^1(\varOmega \backslash \varOmega _r)}\\ =&\frac{1}{r^2}\int _{-1}^{-1+r}\left| -1+r-x\right| \,dx+\frac{1}{r^2}\int _{1-r}^1\left| 1-r-x\right| \,dx\\ =&1, \end{aligned}$$

although

$$\begin{aligned} |\varOmega \backslash \varOmega _r|=2r\underset{r\rightarrow 0}{\rightarrow }0. \end{aligned}$$

Thus,

$$\begin{aligned} {{{\mathcal {A}}}}_ru\underset{r\rightarrow 0}{\rightarrow }0\equiv u' \end{aligned}$$

in the measure but not in $L^1(\varOmega )$.

Example 3.3 supports our idea to average $\nabla u$ instead of *u* itself. The same applies to $\mathring{\nabla }_{r} u$ vs. ${{{\mathcal {S}}}}_r (\nabla u)$.

Averaging w.r.t. $y\in B_1$ and then also w.r.t. $s\in (0,1)$ might appear superfluous in the definition of the operator ${{{\mathcal {T}}}}_r$. The following example compares the effect of ${{{\mathcal {T}}}}_r$ with that of an operator which averages w.r.t. to *y* only.

### Example 3.4

Let $\varOmega =\mathbb {R}^n$, $n\ge 2$, and $r>0$, $F_r\equiv n+1$. In this caseConsider also the operatorIt is easy to see that both operators are well-defined, linear, continuous, and self-adjoint in the space $L^2(\mathbb {R}^n)$. Moreover, they map the dense subspace $C_0(\mathbb {R}^n;\mathbb {R}^n)$ into itself. This suggests the following natural extension to $(C_0(\mathbb {R}^n;\mathbb {R}^n))^*$:

$$\begin{aligned} \langle {{{\mathcal {T}}}}_r\mu ,\varphi \rangle _{(C_0(\mathbb {R}^n;\mathbb {R}^n))^*,C_0(\mathbb {R}^n;\mathbb {R}^n)}:=&\langle \mu ,{{{\mathcal {T}}}}_r\varphi \rangle _{(C_0(\mathbb {R}^n;\mathbb {R}^n))^*,C_0(\mathbb {R}^n;\mathbb {R}^n)},\\ \langle \widetilde{{{{\mathcal {T}}}}}_r\mu ,\varphi \rangle _{(C_0(\mathbb {R}^n;\mathbb {R}^n))^*,C_0(\mathbb {R}^n;\mathbb {R}^n)}:=&\langle \mu ,\widetilde{{{{\mathcal {T}}}}}_r\varphi \rangle _{(C_0(\mathbb {R}^n;\mathbb {R}^n))^*,C_0(\mathbb {R}^n;\mathbb {R}^n)}. \end{aligned}$$

Let, for instance,

$$\begin{aligned} w:=\delta _0e_1, \end{aligned}$$

$\delta _0$ and $e_1$ mean the usual Dirac delta and the vector $(1,0,\dots ,0)$, respectively. One readily computes that

$$\begin{aligned} {\widetilde{{{{\mathcal {T}}}}}}_r(\delta _0 e_1) (x)=\frac{n+1}{|B_{r}|}\chi _{B_{r}}(x)\frac{x_1}{r}\frac{x}{|x|}, \end{aligned}$$

whereas

$$\begin{aligned} {{{\mathcal {T}}}}_r(\delta _0 e_1)(x)&=\frac{n+1}{|B_{r}|}\int _0^1s^{-n-1}\chi _{B_{rs}}(x)\,ds\frac{x_1}{r}\frac{x}{|x|}\\&=\frac{n+1}{n|B_{r}|}\left( \left( \frac{r}{|x|}\right) ^n-1\right) _+\frac{x_1}{r}\frac{x}{|x|}. \end{aligned}$$

For $n\ge 2$, the operator ${{{\mathcal {T}}}}_r$ retains the singularity at the origin, however making it less concentrated, while $\widetilde{{{{\mathcal {T}}}}}_r$ eliminates that singularity entirely and produces instead jump discontinuities all over $S_r$.

### Properties of the averaging operators ${{{\mathcal {T}}}}_r$ and ${{{\mathcal {S}}}}_r$

In this section we collect some properties of the averaging operators ${{{\mathcal {T}}}}_r$ and ${{{\mathcal {S}}}}_r$.

#### Lemma 3.5

(Properties of ${{{\mathcal {T}}}}_r$) Let $F_r$ satisfy Assumptions 1.1 and let $r\in (0,r_0]$. Then:

(i) ${{{\mathcal {T}}}}_r$ is a well-defined continuous linear operator in $(L^{p}(\varOmega ))^n$ for all $p\in [1,\infty ]$. The corresponding operator norm satisfies 3.10 where
(ii) Let $p,p^*\in [1,\infty ]$ be such that $p^*=\frac{p}{p-1}$. For all $w_1\in \left( L^{p}(\varOmega )\right) ^n$ and $w_2\in \left( L^{p^*}(\varOmega )\right) ^n$ it holds:
  3.11 $$\begin{aligned} \int _{\varOmega }({{{\mathcal {T}}}}_rw_1(x)\cdot w_2(x))\,dx=\int _{\varOmega }(w_1(x)\cdot {{{\mathcal {T}}}}_rw_2(x))\,dx. \end{aligned}$$
(iii) Let $p\in [1,\infty )$. For all $w\in (L^{p}(\varOmega ))^n$ it holds that
  3.12 $$\begin{aligned} {{{\mathcal {T}}}}_r w\underset{r\rightarrow 0}{\rightarrow }{{{\mathcal {T}}}}_0w=w\quad \text {in }(L^{p}(\varOmega ))^n. \end{aligned}$$
(iv) For $p =2$ it holds that
  3.13 $$\begin{aligned} \Vert {{{\mathcal {T}}}}_r\Vert _{L((L^2(\varOmega ))^n)} \underset{r\rightarrow 0}{\rightarrow } 1. \end{aligned}$$

#### Remark 3.6

Due to the assumptions on $F_r$ we have in the limit that3.14

#### Proof of Lemma 3.5

(i) Since *w* is measurable and $\rho \mapsto F_r(\rho )$, $(x,s,y)\mapsto x+rsy$, $(y,z)\mapsto (z\cdot y)\frac{y}{|y|}$ are continuous, we have that
  $$\begin{aligned} (x,y,s)\mapsto (w(x+rsy)\cdot y)\frac{y}{|y|}F_r(r|y|) \end{aligned}$$
  is well-defined a.e. in $\varOmega \times B_1\times (0,1)$ and is measurable. Let $p\in {(1},\infty )$ and $p^*=\frac{p}{p-1}$. Using Hölder’s inequality, Fubini’s theorem, and our convention that *w* vanishes outside $\varOmega$, we deduce for all $w\in (L^{p}(\varOmega ))^n$ that  This implies that for all $p\in (1,\infty )$ operator ${{{\mathcal {T}}}}_r$ is well-defined in $(L^{p}(\varOmega ))^n$ and satisfies (3.10). It is also clearly linear. Taken together we then have that ${{{\mathcal {T}}}}_r\in L((L^{p}(\varOmega ))^n)$ and (3.10) holds. The cases $p=1$ and $p=\infty$ can be treated similarly.
(ii) Let $w_1\in \left( L^{p}(\varOmega )\right) ^n$ and $w_2\in \left( L^{p^*}(\varOmega )\right) ^n$. We compute by using Fubini’s theorem, the symmetry of $B_1$, and simple variable transformations that 3.153.16 Thereby we used our convention that each function defined in $\varOmega$ is assumed to be prolonged by zero outside $\varOmega$. Comparing (3.15) and (3.16) we obtain (3.11).
(iii) We apply the Banach–Steinhaus theorem. Due to (i) and (3.14), $\{{{{\mathcal {T}}}}_r\}_{r\in (0,r_0]}$ is a family of uniformly bounded linear operators in the Banach space $(L^{p}(\varOmega ))^n$. Thus, as $C_c({\overline{\varOmega }};\mathbb {R}^n)$ is dense in $(L^{p}(\varOmega ))^n$ for $p<\infty$, we only need to check (3.12) for $w\in C_c({\overline{\varOmega }};\mathbb {R}^n)$. But for such *w* we can directly pass to the limit under the integral and thus obtain using (3.3) and the dominated convergence theorem that
  $$\begin{aligned} {{{\mathcal {T}}}}_r w\underset{r\rightarrow 0}{\rightarrow } {{{\mathcal {T}}}}_0w=w\quad \text {for all }x\in \varOmega { \text { and in }(L^{p}(\varOmega ))^n}. \end{aligned}$$
(iv) Here we make use of the Fourier transform, which we denote by the hat symbol. A straightforward calculation shows that
  $$\begin{aligned} {\widehat{{{{\mathcal {T}}}}_r w}}= \varPhi _r {\widehat{w}}, \end{aligned}$$
  where 3.17 Combining (3.17) with the Plancherel theorem and using our convention that *w* vanishes outside $\varOmega$, we can estimate as follows:
  3.18 $$\begin{aligned} \Vert {{{\mathcal {T}}}}_r\Vert _{L((L^2(\varOmega ))^n)}&= \sup _{\Vert w\Vert _{(L^2(\varOmega ))^n} = 1} \Vert {{{\mathcal {T}}}}_r w\Vert _{(L^2(\varOmega ))^n}\nonumber \\&\le \sup _{\Vert w\Vert _{(L^2(\varOmega ))^n} = 1} \Vert \widehat{{{{\mathcal {T}}}}_r w}\Vert _{(L^2(\mathbb {R}^n))^n}\nonumber \\&\le \Vert {|}\varPhi _r{|}_2 \Vert _{L^{\infty }(\mathbb {R}^n)}\sup _{\Vert w\Vert _{(L^2(\varOmega ))^n} = 1} \Vert {\widehat{w}}\Vert _{(L^2(\mathbb {R}^n))^n}\nonumber \\&= \Vert {|}\varPhi _r{|}_2 \Vert _{L^{\infty }(\mathbb {R}^n)}\sup _{\Vert w\Vert _{(L^2(\varOmega ))^n} = 1} \Vert w\Vert _{(L^2(\varOmega ))^n}\nonumber \\&= \Vert {|}\varPhi _r{|}_2 \Vert _{L^{\infty }(\mathbb {R}^n)}. \end{aligned}$$
  Here $|M|_2$ denotes the spectral norm of a matrix $M\in \mathbb {R}^{n\times n}$. Further, observe that
  3.19 $$\begin{aligned} \varPhi _r(O \xi ) = O \varPhi _r (\xi ) O^T \quad \text { for all orthogonal }O \in \mathbb {R}^{n\times n} \text { and } \xi \in \mathbb {R}^n. \end{aligned}$$
  Consequently, denoting by $e_1$ the first canonical vector of $\mathbb {R}^n$ and appropriately constructing an orthogonal matrix *O* in order for $O\xi =|\xi |e_1$ to hold, we obtain that
  3.20 $$\begin{aligned} {|} \varPhi _r(\xi ) {|}_2 = {|}\varPhi _r(|\xi |e_1){|}_2\quad \text {for all }\xi \in \mathbb {R}^n. \end{aligned}$$
  Since 3.21 is a diagonal matrix, its spectral norm is given by the spectral radius. Estimating the right-hand side of (3.21) we then conclude that 3.22 due to $F_0(0) = n+1$ and (3.4). Combining (3.18),(3.22) and (3.20) we arrive at
  3.23 $$\begin{aligned} \underset{r\rightarrow 0}{\lim \sup }\, \Vert {{{\mathcal {T}}}}_r\Vert _{L((L^2(\varOmega ))^n)}\le 1. \end{aligned}$$
  Finally, the pointwise convergence (3.12) and the Banach–Steinhaus theorem imply that
  $$\begin{aligned} \underset{r\rightarrow 0}{\lim \inf }\, \Vert {{{\mathcal {T}}}}_r\Vert _{L((L^2(\varOmega ))^n)}\ge 1, \end{aligned}$$
  concluding the proof.

$$\square $$

A similar result holds for ${{{\mathcal {S}}}}_r$:

#### Lemma 3.7

(Operator ${{{\mathcal {S}}}}_r$) Let $r\in [0,r_0]$. Then:

(i) ${{{\mathcal {S}}}}_r$ is a well-defined continuous linear operator in $(L^{p}(\varOmega ))^n$ for all $p\in [1,\infty ]$. The corresponding operator norm satisfies
  3.24 $$\begin{aligned} \Vert {{{\mathcal {S}}}}_r\Vert _{L((L^{p}(\varOmega ))^n)}\le n. \end{aligned}$$
(ii) Let $p,p^*\in [1,\infty ]$ be such that $p^*=\frac{p}{p-1}$. For all $w_1\in \left( L^{p}(\varOmega )\right) ^n$ and $w_2\in \left( L^{p^*}(\varOmega )\right) ^n$ it holds:
  $$\begin{aligned} \int _{\varOmega }({{{\mathcal {S}}}}_rw_1(x)\cdot w_2(x))\,dx=\int _{\varOmega }(w_1(x)\cdot {{{\mathcal {S}}}}_rw_2(x) )\,dx. \end{aligned}$$
(iii) Let $p\in [1,\infty )$. For all $w\in (L^p(\varOmega ))^n$ it holds that
  $$\begin{aligned} {{{\mathcal {S}}}}_r w\underset{r\rightarrow 0}{\rightarrow } {{{\mathcal {S}}}}_0w=w\quad \text {in }(L^{p}(\varOmega ))^n. \end{aligned}$$
(iv) For $p =2$ it holds that
  $$\begin{aligned} \Vert {{{\mathcal {S}}}}_r\Vert _{L((L^2(\varOmega ))^n)} \underset{r\rightarrow 0}{\rightarrow } 1. \end{aligned}$$

#### Proof

The proof almost repeats that of Lemma 3.5. Therefore, we only check (3.24) and omit further details. Let $p\in [1,\infty )$ and $p^*=\frac{p}{p-1}$. Using Hölder’s inequality, Fubini’s theorem, and our convention that *w* vanishes outside $\varOmega$ we deduce for all $w\in (L^{p}(\varOmega ))^n$ thatwhich means that

3.25 $$\begin{aligned} \Vert {{{\mathcal {S}}}}_r\Vert _{L((L^{p}(\varOmega ))^n)}\le n. \end{aligned}$$

The proof in the case $p=\infty$ follows the same steps, or, alternatively, one passes to the limit as $p\rightarrow \infty$ in (3.25). $\square$

#### Remark 3.8

The constants in (3.10) for any $n\ge 1$ and in (3.24) for $n\ge 2$ are not necessarily optimal. For $p \ne 2$ it remains open whether or not

$$\begin{aligned}&\underset{r\rightarrow 0}{\lim \inf }\left\| {{{\mathcal {T}}}}_{r}\right\| _{ L((L^{ p}(\varOmega ))^n)}=1,\\&\underset{r\rightarrow 0}{\lim \inf }\left\| {{{\mathcal {S}}}}_{r}\right\| _{ L((L^{ p}(\varOmega ))^n)}=1. \end{aligned}$$

The answer may depend upon $\varOmega$ and *p*.

## Well-posedness for a class of evolution equations involving ${{{\mathcal {T}}}}_r$ or ${{{\mathcal {S}}}}_r$

In this section we establish the existence and uniqueness of solutions to a certain class of single evolution equations involving ${{{\mathcal {T}}}}_r$ or ${{{\mathcal {S}}}}_r$. This result is an important ingredient for our analysis of nonlocal systems in Sect. 5. Thus, we consider the following initial boundary value problem: 4.1a4.1b4.1c Here

$$\begin{aligned} {{{\mathcal {R}}}}_r\in \{{{{\mathcal {T}}}}_r,{{{\mathcal {S}}}}_r\}, \end{aligned}$$

and for $\varepsilon \ge 0$ we set

4.2 $$\begin{aligned} G_{\varepsilon }: \mathbb {R}^n \rightarrow \mathbb {R}^n, \quad x \mapsto \frac{x}{1+ \varepsilon |x|}. \end{aligned}$$

A standard calculation shows that $G_{\varepsilon }$ is globally Lipschitz with a Lipschitz constant 1.

### Remark 4.1

Observe that for $\varepsilon =0$ equation (4.1a) is linear, whereas for $\varepsilon >0$ the nonlocal part of the flux is a priori bounded. The latter helps us to construct nonnegative solutions in Sect. 5.

We make the following assumptions:4.34.44.54.64.7To shorten the notation, we introduce a pair of constantsDue to assumptions (4.3)–(4.5) it is clear that

$$\begin{aligned} {0<}\alpha _r, M_r{<\infty .} \end{aligned}$$

We introduce a family of operators

### Lemma 4.2

Let (4.3)–(4.5) be satisfied. Then:

(i) For a.a. $t\in [0,T]$ the operator
  $$\begin{aligned} {\mathcal {M}}(t,\cdot ):H^1(\varOmega ) \rightarrow (H^1(\varOmega ))^* \end{aligned}$$
  is well-defined, monotone, hemicontinuous, and satisfies the bounds
  4.8 $$\begin{aligned}&\langle {{{\mathcal {M}}}}(t,u),u \rangle _{{(H^1(\varOmega ))^*,H^1(\varOmega )}} \ge {\alpha _{r} ||\nabla u ||_{(L^2(\varOmega ))^n}^{{2}}}, \end{aligned}$$
  4.9 $$\begin{aligned}&||{{{\mathcal {M}}}}(t,u)||_{(H^1(\varOmega ))^*} \le M_r ||\nabla u ||_{(L^2(\varOmega ))^n} \end{aligned}$$
  for all $u\in H^1(\varOmega )$. Moreover, for all $u\in H^1(\varOmega )$ the function ${\mathcal {M}}(\cdot ,u)$ is measurable.
(ii) The operator
  $$\begin{aligned} {\mathcal {M}}:L^2(0,T;H^1(\varOmega )) \rightarrow L^2(0,T;(H^1(\varOmega ))^* ) \end{aligned}$$
  is well-defined, monotone, hemicontinuous, and satisfies the bounds
  $$\begin{aligned}&\langle {{{\mathcal {M}}}}(u),u \rangle _{{ L^2(0,T;(H^1(\varOmega ))^*),L^2(0,T;H^1(\varOmega ))}} \ge {\alpha _{r} ||\nabla u ||_{L^2(0,T;(L^2(\varOmega ))^n)}^{{ 2}}},\\&||{{{\mathcal {M}}}}(u)||_{L^2(0,T;(H^1(\varOmega ))^* )} \le M_r ||\nabla u ||_{L^2(0,T;(L^2(\varOmega ))^n)} \end{aligned}$$
  for all $$u\in L^2(0,T;H^1(\varOmega ))$$.

### Proof

The assumptions on the coefficients $a_i$ together with the Lipschitz continuity of $G_{\varepsilon }$ readily imply that for a.a. $t\in [0,T]$ the operator ${{{\mathcal {M}}}}(t, \cdot )$ is well-defined and satisfies (4.9). Moreover, due to (4.3) and $G_{\varepsilon }$ Lipschitz, it is also clear that ${{{\mathcal {M}}}}(\cdot ,u):[0,T]\rightarrow (H^1(\varOmega ))^*$ is measurable on [0, *T*] for all $u\in H^1(\varOmega )$, whereas for a.a. *t* the mapping $\lambda \mapsto \langle {{{\mathcal {M}}}}(t,u + \lambda v), w\rangle _{{(H^1(\varOmega ))^*,H^1(\varOmega )}}$ is continuous on $\mathbb {R}$, the latter meaning that ${\mathcal {M}}(t,\cdot )$ is hemicontinuous. Using Hölder’s inequality, the fact that $G_{\varepsilon }$ is Lipschitz with Lipschitz constant 1, the assumptions on the $a_i$’s, and the properties of ${{{\mathcal {R}}}}_r$, we compute that4.10for $u,v\in H^1(\varOmega )$, which proves monotonicity. Further, taking $v = 0$ in (4.10) and using ${{{\mathcal {M}}}}(t,0) = 0$ yields (4.8). Part (i) is thus proved. A proof of (ii) can be done similarly; we omit further details. $\square$

Using the properties of the averaging operators proved in Sect. 3.1 we can define weak solutions to (4.1) in a manner very similar to that for the classical, purely local case (i.e., when ):

### Definition 4.3

Let (4.3)–(4.7) hold. We call the function $c_r:[0,T]\times {\overline{\varOmega }}\rightarrow \mathbb {R}$ a weak solution of (4.1) if:

(i) $$c_r\in L^2(0,T;H^1(\varOmega ))\cap C([0,T];L^2(\varOmega ))$$, $$\partial _t c_r\in L^2(0,T;(H^1(\varOmega ))^*)$$;
(ii) $c_r$ satisfies (4.1a)–(4.1b) in the following sense: for all $\varphi \in H^1(\varOmega )$ and a.a. $t\in (0,T)$4.11
(iii) $$c_r(0,\cdot )=c_0$$ in $$L^2(\varOmega )$$.

Using standard theory one readily proves the following existence result:

### Lemma 4.4

Let (4.3)–(4.7) hold. Then there exists a unique weak solution to (4.1) in terms of Definition 4.3. The solution satisfies the following estimates:4.124.13

### Proof

The existence of a unique weak solution to (4.1) is a direct consequence of Lemma 4.2(i) and the standard theory of evolution equations with monotone operators, see, e.g. Showalter (1997), Chapter III Proposition 4.1). It remains to check the bounds (4.12), and (4.13). Taking $\varphi :=c_r$ in the weak formulation (4.11) and using (Temam 2001, Chapter III Lemma 1.2), (4.8), and the Young inequality, we obtain that

$$\begin{aligned} \frac{1}{2}\frac{d}{dt}\Vert c_r\Vert _{L^2(\varOmega )}^2&\le -\alpha _r\Vert \nabla c_r\Vert _{(L^2(\varOmega ))^n}^2+\Vert c_r\Vert _{H^1(\varOmega )}\Vert f\Vert _{(H^1(\varOmega ))^*}\\&=-\alpha _r\Vert c_r\Vert _{H^1(\varOmega )}^2+\alpha _r\Vert c_r\Vert _{L^2(\varOmega )}^2+\Vert c_r\Vert _{H^1(\varOmega )}\Vert f\Vert _{(H^1(\varOmega ))^*}\\&\le -\frac{1}{2}\alpha _r\Vert c_r\Vert _{H^1(\varOmega )}^2+\alpha _r\Vert c_r\Vert _{L^2(\varOmega )}^2+\frac{1}{2}\alpha _r^{-1}\Vert f\Vert _{(H^1(\varOmega ))^*}^2, \end{aligned}$$

which yields (4.12) due to the Gronwall lemma. Finally, using (4.9), we obtain from the weak formulation (4.11) that

$$\begin{aligned} \Vert \partial _t c_r\Vert _{L^2(0,T;(H^1(\varOmega ))^*)}^2\le&2M_r^2\Vert \nabla c_r\Vert _{L^2(0,T;(L^2(\varOmega ))^n)}^2+2\Vert f\Vert _{L^2(0,T;(H^1(\varOmega ))^*)}^2. \end{aligned}$$

Together with (4.12) this implies (4.13). $\square$

## Nonlocal models involving averaging operators ${{{\mathcal {T}}}}_r$ and ${{{\mathcal {S}}}}_r$

In this section we study the following model IBVP:

5.1a $$\begin{aligned}&\partial _t c_{r}=\nabla \cdot \left( D_c(c_{r},v_{r})\nabla c_{r }-c_{r}\chi (c_{r},v_{r}) {{{\mathcal {R}}}}_{r}(\nabla g(c_r,v_r))\right) +f_c(c_{r},v_{r})&\text { in }\mathbb {R}^+\times \varOmega ,\! \end{aligned}$$

5.1b $$\begin{aligned}&\partial _t v_{r}=D_v\varDelta v_r+f_v(c_{r},v_{r})&\text { in }\mathbb {R}^+\times \varOmega ,\! \end{aligned}$$

5.1c $$\begin{aligned}&D_c(c_{r},v_{r})\partial _{\nu } c_{r }-c_{r}\chi (c_{r},v_{r}) {{{\mathcal {R}}}}_{r}(\nabla g(c_r,v_r))\cdot \nu =D_v \partial _{\nu }v_{r}=0&\!\!\!\text { in }\mathbb {R}^+\times \partial \varOmega , \end{aligned}$$

5.1d $$\begin{aligned}&c_{r}(0,\cdot )=c_0,\ v_{r}(0,\cdot )=v_0&\text { in }\varOmega . \end{aligned}$$

Here, as in the previous section, ${{{\mathcal {R}}}}_r$ stands for any of the two averaging operators:

$$\begin{aligned} {{{{\mathcal {R}}}}_r\in \{{{{\mathcal {T}}}}_r,{{{\mathcal {S}}}}_r\}.} \end{aligned}$$

We assume that the diffusion coefficient $D_v$ is either a positive number, or it is zero.

Equations (5.1a)–(5.1b) are closely related to (1.1) and (1.4) in Sect. 1, the difference being that the terms involving the adhesion velocity/non-local gradient are now replaced by those including the averaging operators ${{{\mathcal {T}}}}_{r}$/${{{\mathcal {S}}}}_{r}$ from Sect. 3. Our motivation for introducing this change is twofold. First of all, due to (3.5) and (3.8) it affects the points in the boundary layer $\varOmega \backslash \varOmega _r$, at the most. On the other hand, Example 3.3 indicates that including, e.g., ${{{\mathcal {A}}}}_r$ can lead to limits with unexpected blow-ups on the boundary of $\varOmega$.

System (5.1) is a non-local version of the hapto-/chemotaxis system

5.2a $$\begin{aligned}&\partial _t c=\nabla \cdot \left( D_c(c,v)\nabla c-c\chi (c,v) \nabla g(c,v)\right) +f_c(c,v)&\quad \text { in }\mathbb {R}^+\times \varOmega , \end{aligned}$$

5.2b $$\begin{aligned}&\partial _t v=D_v\varDelta v+f_v(c,v)&\quad \text { in }\mathbb {R}^+\times \varOmega , \end{aligned}$$

5.2c $$\begin{aligned}&D_c(c,v)\partial _{\nu } c_{r }-c\chi (c,v) \partial _{\nu } g(c,v)=D_v \partial _{\nu }v=0&\quad \text { in }\mathbb {R}^+\times \partial \varOmega , \end{aligned}$$

5.2d $$\begin{aligned}&c(0,\cdot )=c_0,\ v(0,\cdot )=v_0&\quad \text { in }\varOmega . \end{aligned}$$

In this case, the actual diffusion and haptotactic sensitivity coefficients are

$$\begin{aligned} {{\widetilde{D}}}_c(c,v)&=D_c(c,v)-c\chi (c,v)\partial _cg(c,v),\\ {{\widetilde{\chi }}}(c,v)&=\chi (c,v)\partial _vg(c,v), \end{aligned}$$

so that in the classical formulation (5.2a) takes the form

$$\begin{aligned} \partial _t c=\nabla \cdot \left( {{\widetilde{D}}}_c(c,v)\nabla c-c{{\widetilde{\chi }}}(c,v) \nabla v\right) +f_c(c,v).\quad \text { in }\mathbb {R}^+\times \varOmega . \end{aligned}$$

The main goal of this section is to establish, under suitable assumptions on the system coefficients which are introduced in Sect. 5.1, a rigorous convergence as $r\rightarrow 0$ of solutions of the nonlocal model family (5.1) to those of the local model (5.2), see Theorem 5.8. This is accomplished in the final Sect. 5.4. Since we are dealing here with a new type of nonlocal system, we establish for (5.1) the existence of nonnegative solutions in Sects. 5.2, and 5.3.

### Problem setting and main result of the section

We begin with several general assumptions about the coefficients of system (5.1).

#### Assumptions 5.1

Let $D_v\in \mathbb {R}^+_0$, $D_c,\chi \in C_b(\mathbb {R}^+_0\times \mathbb {R}^+_0)$, and $g,f_c,f_v\in C^1(\mathbb {R}^+_0\times \mathbb {R}^+_0)$:Assume that the coefficients satisfy the following bounds:5.35.4Further, we assume that the initial values satisfy

5.5 $$\begin{aligned}&0\le c_0 \in L^2(\varOmega ),\nonumber \\&{0\le v_0} \in H^1(\varOmega ). \end{aligned}$$

#### Remark 5.2

If $D_v>0$, then assumption (5.5) can be replaced by a weaker one, such as

$$\begin{aligned} v_0\in L^2(\varOmega ). \end{aligned}$$

We keep (5.5) in order to simplify the exposition.

In addition, we will later choose one of the following assumptions on $f_c$ and the nonlocal operator:

#### Assumptions 5.3

(Further assumptions on $f_c$) One of the following conditions holds:

- $$\begin{aligned} \nabla _{(c,v)}f_c \in \left( L^{\infty }(\mathbb {R}_0^+\times \mathbb {R}_0^+)\right) ^2 \end{aligned}$$
- there exists $s\ge 0$ such that 5.6

#### Assumptions 5.4

(Assumptions on ${\mathcal {R}}_r$) One of the following holds:

- for a given fixed $r\in (0,r_0]$
- 5.7

#### Example 5.5

Let

$$\begin{aligned} D_v&=0,\\ F_r(\rho )&:=(n+1)e^{-r\rho },\\ g(c,v)&:=\frac{S_{cc}c+S_{cv}v}{1+c+v}\quad \text { for some constants}\quad S_{cc},S_{cv}>0,\\ D_c(c,v)&:= \frac{1+c}{1+c+v},\\ \chi (c,v)&:=\frac{b}{1+c+v},\quad b>0,\\ f_c(c,v)&:=\mu _c{\frac{c}{1+c^2}}(K_c-c-\eta _c v)\quad \text { for some constants}\ K_c,\eta _c>0,\ \mu _c {>} 0,\\ f_v(c,v)&:=\mu _vv(K_v-v)-\lambda _vv{\frac{c}{1+c}}\ \ \text { for some constants}\ K_v,\lambda _v>0,\ \mu _v\ge 0, \end{aligned}$$

and assume that

$$\begin{aligned} 0\le v_0\le K_v. \end{aligned}$$

Then, it holds a priori that

$$\begin{aligned} 0\le v\le K_v \end{aligned}$$

for any *v* which solves (5.1b). Therefore it suffices to consider the coefficient functions in $\mathbb {R}_0^+ \times [0,K_v]$.

For $D_c$ it holds on $\mathbb {R}_0^+ \times [0,K_v]$ thatandMoreover, $\nabla _{(c,v)}g,\ \nabla _{(c,v)} f_v \in (L^{\infty }(\mathbb {R}^+_0\times \mathbb {R}^+_0))^2$, due toand

$$\begin{aligned} \sup _{c,v \ge 0} |\partial _v f_v(c,v)| = \sup _{c,v \ge 0}\left| \mu _v(K_v-2v)-\lambda _v \frac{c}{1+c} \right| < \infty . \end{aligned}$$

For ,  and  we can estimate on $\mathbb {R}^+_0\times \mathbb {R}^+_0$ thatFurther,holds.

Thus, Assumptions 5.1, 5.3(b) and 5.4 (b) are fulfilled if

$$\begin{aligned} (1+K_v)b\max \left\{ S_{cc},\left| \frac{S_{cc}}{1+K_v}-\frac{S_{cv}K_v}{(1+K_v)^2}\right| \right\} < 1. \end{aligned}$$

This choice of coefficient functions can be used to describe a population of cancer cells which interact among themselves and with the surrounding extracellular matrix (ECM) tissue. Both interaction types are due to adhesion, whether to other cells (cell–cell adhesion) or to the matrix (cell–matrix adhesion). The interaction force $F_r(\rho )$ is taken to diminish with increasing interaction range $\rho$ and/or of the sensing radius *r*: cells too far apart/out of reach hardly interact in a direct way. Function *g*(*c*, *v*) characterises effective interactions. Here the coefficients $S_{cc}$ and $S_{cv}$ represent cell–cell and cell–matrix adhesion strengths, respectively. Our choice of *g* accounts for some adhesiveness limitation imposed by high local cell and tissue densities. It is motivated by the fact that overcrowding may preclude further adhesive bonds, e.g. due to saturation of receptors. The diffusion coefficient $D_c(c,v)$ is chosen to be everywhere positive and increase with a growing population density, thus enhancing diffusivity under population pressure, but, further, limited by excessive cell–tissue interaction. The latter also applies to the choice of the sensitivity function $\chi$. Indeed, there is evidence that tight packing of cells and ECM limits diffusivity and the advective effects of haptotaxis (Lu et al. 2012). Thereby the constant $b>0$ is assumed to be rather small. Finally, $f_c$ and $f_v$ describe growth of cells and tissue limited by concurrence for resources.

Next, we introduce weak-strong solutions to our problem. The definition is as follows:

#### Definition 5.6

Let Assumptions 5.1 hold. Let $r\in [0,r_0]$. We call a pair of functions $(c_r,v_r):\mathbb {R}^+_0\times {\overline{\varOmega }}\rightarrow \mathbb {R}^+_0\times \mathbb {R}^+_0$ a global weak-strong solution of (5.1) if for all $T>0$:

(i) $$c_r\in L^2(0,T;H^1(\varOmega ))\cap C_w([0,T];L^2(\varOmega ))$$, $$\partial _t c_r\in L^1(0,T;(W^{1,\infty }(\varOmega ))^*)$$;
(ii) $$v_r\in C([0,T];H^1(\varOmega ))$$, $$\partial _t v_r\in L^2(0,T;L^2(\varOmega ))$$, $$D_v v_r\in L^2(0,T;H^2(\varOmega ))$$;
(iii) $$f_c(c_r,v_r)\in L^1(0,T;L^1(\varOmega ))$$, $$f_v(c_r,v_r)\in L^{ 2}(0,T;L^{ 2}(\varOmega ))$$;
(iv) $(c_r,v_r)$ satisfies (5.1) in the following weak-strong sense: for all $\varphi \in C^1({\overline{\varOmega }})$ and a.a. $t\in (0,T)$
  5.8a $$\begin{aligned}&\langle \partial _t c_r,\varphi \rangle _{{(W^{1,\infty }(\varOmega ))^*,W^{1,\infty }(\varOmega )}}\nonumber \\ =&-\int _{\varOmega }\left( D_c(c_{r},v_{r})\nabla c_{r }-c_{r}\chi (c_{r},v_{r}) {{{\mathcal {R}}}}_{r}(\nabla g(c_r,v_r))\right) \cdot \nabla \varphi \, dx\nonumber \\&+ \int _{\varOmega }f_c(c_r,v_r)\varphi \, dx, \end{aligned}$$
  5.8b $$\begin{aligned}&c_{{r}}(0,\cdot )=c_0\quad \text {in }L^2(\varOmega ), \end{aligned}$$
  and
  5.8c $$\begin{aligned}&{\partial _t v_r=D_v\varDelta v_r+f_v(c_r,v_r)}\quad&{ \text {a.e. in }(0,T)\times \varOmega }, \end{aligned}$$
  5.8d $$\begin{aligned}&{ D_v \partial _{\nu } v_r=0\quad }&{ \text {a.e. in }(0,T)\times \partial \varOmega }, \end{aligned}$$
  5.8e $$\begin{aligned}&v_{{r}}(0,\cdot )=v_0\quad&\text {in }H^1(\varOmega ). \end{aligned}$$

#### Remark 5.7

Observe that for $r=0$ we obtain a corresponding solution definition for the local system (5.2).

Our main result now reads:

#### Theorem 5.8

Let Assumptions 1.1, 5.1, 5.3, and 5.4(b) hold. Then, there exists a sequence $r_m\rightarrow 0$ as $m\rightarrow \infty$ and solutions $(c_{r_m},v_{r_m})$ and (*c*, *v*) in terms of Definition 5.6 corresponding to $r=r_m$ and $r=0$, respectively, s.t.

$$\begin{aligned} c_{r_m}&\underset{m \rightarrow \infty }{\rightarrow } c \quad \text {in } L^2(0,T;L^2(\varOmega )) ,\\ v_{r_m}&\underset{m \rightarrow \infty }{\rightarrow } v \quad \text {in } L^2(0,T;L^2(\varOmega )). \end{aligned}$$

This Theorem is proved in Sect. 5.4.

#### Notation 5.9

Dependencies upon such parameters as the space dimension *n*, domain $\varOmega$, function *c*, the norms of the initial data $c_0$ and $v_0$, norms and bounds for the coefficient functions are mostly **not** indicated in an explicit way.

### Global existence of solutions to (5.1): the case of $f_c$ Lipschitz

In this subsection we address the existence of solutions to the nonlocal model (5.1) for the case when $f_c$ satisfies Assumptions 5.3(a). The main result of the subsection is as follows:

#### Theorem 5.10

Let Assumptions 1.1, 5.1, and 5.3(a) hold and let *r* satisfy Assumption 5.4(a). Then there exists a global weak-strong solution to (5.1) in terms of Definition 5.6 with $\partial _t c_r\in L^2(0,T;(H^1(\varOmega ))^*)$.

Since we aim at constructing nonnegative solutions, it turns out to be helpful to consider first the following family of approximating problems:

5.9a $$\begin{aligned}&\partial _t c_{r\varepsilon }=\nabla \cdot \left( D_c(c_{r\varepsilon },v_{r\varepsilon })\nabla c_{r\varepsilon }-c_{r\varepsilon }\chi (c_{r\varepsilon },v_{r\varepsilon })\Big ( G_{\varepsilon }( {{{\mathcal {R}}}}_{r}(\partial _c g(c_{r\varepsilon },v_{r\varepsilon }) \nabla c_{r\varepsilon }))\right. \nonumber \\&\quad \left. + G_{\varepsilon }( {{{\mathcal {R}}}}_{r}(\partial _v g(c_{r\varepsilon },v_{r\varepsilon }) \nabla v_{r\varepsilon }))\Big )\right) +f_c(c_{r\varepsilon },v_{r\varepsilon })&\!\!\!\!\!\!\!\!\!\!\!\!\!\!\!\!\!\!\!\!\!\!\text { in }\mathbb {R}^+\times \varOmega , \end{aligned}$$

5.9b $$\begin{aligned}&\partial _t v_{r\varepsilon }=D_v\varDelta v_{r\varepsilon }+f_v(c_{r\varepsilon },v_{r\varepsilon })&\!\!\!\!\!\!\!\!\!\!\!\!\!\!\!\!\!\!\!\!\!\!\text { in }\mathbb {R}^+\times \varOmega , \end{aligned}$$

5.9c $$\begin{aligned}&D_c(c_{r\varepsilon },v_{r\varepsilon })\nabla c_{r\varepsilon }-c_{r\varepsilon }\chi (c_{r\varepsilon },v_{r\varepsilon })\Big ( G_{\varepsilon }( {{{\mathcal {R}}}}_{r}(\partial _c g(c_{r\varepsilon },v_{r\varepsilon }) \nabla c_{r\varepsilon })) \nonumber \\&\quad + G_{\varepsilon }( {{{\mathcal {R}}}}_{r}(\partial _v g(c_{r\varepsilon },v_{r\varepsilon }) \nabla v_{r\varepsilon }))\Big )\cdot \nu =D_v \partial _{\nu }v_{r\varepsilon }=0&\!\!\!\!\!\!\!\!\!\!\!\!\!\!\!\!\!\!\!\!\!\!\text { in }\mathbb {R}^+\times \partial \varOmega , \end{aligned}$$

5.9d $$\begin{aligned}&c_{r\varepsilon }(0,\cdot )=c_0,\ v_{r\varepsilon }(0,\cdot )=v_0&\!\!\!\!\!\!\!\!\!\!\!\!\!\!\!\!\!\!\!\!\!\!\text { in }\varOmega , \end{aligned}$$

where $G_{\varepsilon }$ was defined in (4.2). In order to obtain existence for the original problem, i.e., for $\varepsilon =0$, we first prove existence of nonnegative solutions for the cases when $\varepsilon , D_c>0$. This corresponds to a chemotaxis problem with a nonlocal flux-limited drift. Weak-strong solutions to (5.9) are understood as in Definition 5.6, with the obvious modification of the weak formulation, which now reads:

5.10 $$\begin{aligned}&\langle \partial _t c_{r\varepsilon },\varphi \rangle _{{(H^1(\varOmega ))^*,H^1(\varOmega )}}\nonumber \\&\quad =-\int _{\varOmega }{D_c(c_{r\varepsilon },v_{r\varepsilon })}\nabla c_{r\varepsilon }\cdot \nabla \varphi \, dx\nonumber \\&\quad + \int _{\varOmega }{ c_{r\varepsilon }\chi (c_{r\varepsilon },v_{r\varepsilon })} G_{\varepsilon }({{{\mathcal {R}}}}_{r}(\partial _cg(c_{r\varepsilon },v_{r\varepsilon })\nabla c_{r\varepsilon }))\cdot \nabla \varphi \,dx\nonumber \\&\quad +\int _{\varOmega }{c_{r\varepsilon }\chi (c_{r\varepsilon },v_{r\varepsilon })}G_{\varepsilon }({{{\mathcal {R}}}}_{r} (\partial _vg(c_{r\varepsilon },v_{r\varepsilon })\nabla v_{r\varepsilon })) \cdot \nabla \varphi +f_c(c_{r\varepsilon },v_{r\varepsilon })\varphi \, dx. \end{aligned}$$

#### Lemma 5.11

Let Assumptions of Theorem 5.10 be satisfied. Assume further that

$$\begin{aligned} \varepsilon , D_v>0. \end{aligned}$$

Then there exists a global weak-strong solution to (5.9) with

$$\begin{aligned} \partial _tc_{r\varepsilon }\in L^2(0,T;(H^1(\varOmega ))^*). \end{aligned}$$

#### Proof

To begin with, we extend the coefficients: for $c<0$

$$\begin{aligned}&(D_c,\chi )(c,v) := (D_c, \chi )(-c,v), \quad f_c(c,v) := -f_c (-c,v),\\&g(c,v) := 2g(0,v)-g(-c,v), \quad f_v(c,v) := 2f_v(0,v)-f_v(-c,v). \end{aligned}$$

These coefficients still satisfy Assumptions 5.1, 5.3(a), and 5.4(a) if we consider all suprema over $c \in \mathbb {R}$ instead of $c \in \mathbb {R}_0^+$.

Our approach to proving existence is based on the classical Leray-Schauder principle (Zeidler 1986, Chapter 6, §6.8, Theorem 6.A). In order to apply this theorem we first ’freeze’ $c_{r\varepsilon }$ in the system coefficients of (5.9), replacing it by ${{\bar{c}}}_{r\varepsilon }$. Correspondingly, we obtain the following weak formulation in place of (5.10): For all $\varphi \in H^1(\varOmega )$ and a.a. $t>0$

5.11a $$\begin{aligned}&\langle \partial _t c_{r\varepsilon },\varphi \rangle _{{(H^1(\varOmega ))^*,H^1(\varOmega )}}\nonumber \\&\quad =-\int _{\varOmega }D_c({\bar{c}}_{r\varepsilon },v_{r\varepsilon })\nabla c_{r\varepsilon }\cdot \nabla \varphi \, dx\nonumber \\&\quad + \int _{\varOmega }{\bar{c}}_{r\varepsilon }\chi ({\bar{c}}_{r\varepsilon },v_{r\varepsilon }) G_{\varepsilon }({{{\mathcal {R}}}}_{r}(\partial _cg({\bar{c}}_{r\varepsilon },v_{r\varepsilon })\nabla c_{r\varepsilon }))\cdot \nabla \varphi \,dx\nonumber \\&\quad +\int _{\varOmega }{\bar{c}}_{r\varepsilon }\chi ({\bar{c}}_{r\varepsilon },v_{r\varepsilon })G_{\varepsilon }({{{\mathcal {R}}}}_{r} (\partial _vg({\bar{c}}_{r\varepsilon },v_{r\varepsilon })\nabla v_{r\varepsilon })) \cdot \nabla \varphi +f_c({\bar{c}}_{r\varepsilon },v_{r\varepsilon })\varphi \, dx, \end{aligned}$$

5.11b $$\begin{aligned}&c_{{r\varepsilon }}(0,\cdot )=c_0\quad \text {in }L^2(\varOmega ) \end{aligned}$$

and

5.11c $$\begin{aligned}&\partial _t v_{r\varepsilon }=D_v\varDelta v_{r\varepsilon }+f_v({\bar{c}}_{r\varepsilon },v_{r\varepsilon })\quad&\text {a.e. in }(0,T)\times \varOmega , \end{aligned}$$

5.11d $$\begin{aligned}&D_v \partial _{\nu } v_{r\varepsilon }=0&\text {a.e. in }(0,T)\times \partial \varOmega , \end{aligned}$$

5.11e $$\begin{aligned}&v_{r\varepsilon }(0,\cdot )=v_0\quad&\text {in }H^1(\varOmega ). \end{aligned}$$

Let $T>0$ and let ${\bar{c}}_{r\varepsilon }\in L^2(0,T;L^2(\varOmega ))$. Since $f_v$ is assumed to be Lipschitz, we can make use of the standard theory (Ladyzhenskaya et al. 1968) which implies that the semilinear parabolic initial boundary value problem (5.11c)–(5.11e) possesses a unique global strong solution $0\le v_{r\varepsilon }\in L^2(0,T;H^2(\varOmega ))$ with $\partial _t v_{r\varepsilon }\in L^2(0,T;L^2(\varOmega ))$, and satisfying the estimate5.12Here and further in the proof we omit the dependence of constants upon $D_v$. SetDue to our assumptions about $D_c,\chi ,g$, and $f_c$, these coefficients $a_i$ and *f* satisfy the requirements of Lemma 4.2. Consequently, there exists a unique global weak solution $c_{r\epsilon }$ to problem (4.1) with these coefficients. We estimate for the corresponding constants $\alpha _r$ and $M_r$ introduced in Lemma 4.2:5.135.14and, due to (5.12),5.15Combining (4.12)–(4.13) and (5.13)–(5.15), we obtain the following bounds for $c_{ r\epsilon }$:5.165.17Now consider the mapping

$$\begin{aligned} \varPhi :{{\bar{c}}_{r\varepsilon }}\mapsto c_{r\varepsilon }. \end{aligned}$$

Thanks to (5.16) and (5.17), $\varPhi$ is well-defined in $L^2(0,T;L^2(\varOmega ))$ and

5.18 $$\begin{aligned}&\varPhi :L^2(0,T;L^2(\varOmega ))\rightarrow \{ u\in L^2(0,T;H^1(\varOmega )):\ \partial _t u\in L^2(0,T;(H^1(\varOmega ))^*)\} \nonumber \\&\text {maps bounded sets on bounded sets}. \end{aligned}$$

Due to the Lions–Aubin lemma, (5.18) implies that

5.19 $$\begin{aligned}&\varPhi :L^2(0,T;L^2(\varOmega ))\rightarrow L^2(0,T;L^2(\varOmega )) \end{aligned}$$

5.20 $$\begin{aligned}&\text {maps bounded sets on precompact sets}. \end{aligned}$$

Next, we verify that $\varPhi$ is closed in $L^2(0,T;L^2(\varOmega ))$. Consider a sequence

$$\begin{aligned} \{ {\bar{c}}_{r\varepsilon m}\}\subset L^2(0,T;L^2(\varOmega )) \end{aligned}$$

s.t.

5.21 $$\begin{aligned}&{{\bar{c}}_{r\varepsilon m}}\underset{m\rightarrow \infty }{\rightarrow } {{\bar{c}}_{r\varepsilon }}\quad \text {in }L^2(0,T;L^2(\varOmega )), \end{aligned}$$

5.22 $$\begin{aligned} \varPhi ({{\bar{c}}_{r\varepsilon m}})=:&{ c_{r\varepsilon m}}\underset{m\rightarrow \infty }{\rightarrow }{c_{r\varepsilon }}\quad \text {in }L^2(0,T;L^2(\varOmega )). \end{aligned}$$

We need to check that

$$\begin{aligned} \varPhi ( {{\bar{c}}_{r\varepsilon }}) = {c_{r\varepsilon }}. \end{aligned}$$

Due to (5.21) we have (by switching to a subsequence, if necessary) that

5.23 $$\begin{aligned} {{\bar{c}}_{r\varepsilon m}}\underset{m\rightarrow \infty }{\rightarrow } {{\bar{c}}_{r\varepsilon }}\quad \text {a.e.} \end{aligned}$$

Further, (5.18) and (5.22) together with the Banach–Alaoglu theorem imply that

5.24 $$\begin{aligned}&{{ c_{r\varepsilon m}}\underset{m\rightarrow \infty }{\rightharpoonup } { c_{r\varepsilon }}}&\quad {\text {in }L^2(0,T;H^1(\varOmega ))}, \end{aligned}$$

5.25 $$\begin{aligned}&\partial _t { c_{r\varepsilon m}}\underset{m\rightarrow \infty }{\rightharpoonup } \partial _t{ c_{r\varepsilon }}&\quad \text {in }L^2(0,T;(H^1(\varOmega ))^*). \end{aligned}$$

By the definition of $\varPhi$ we have that ${\bar{c}}_{r\varepsilon m}$ and $c_{r\varepsilon m}$ satisfy: for all $\varphi \in H^1(\varOmega )$ and a.a. $t\in (0,T)$

5.26a $$\begin{aligned}&\langle \partial _t {c_{r\varepsilon m}},\varphi \rangle _{{(H^1(\varOmega ))^*,H^1(\varOmega )}}\nonumber \\&\quad =-\int _{\varOmega }{ D_c({ {\bar{c}}_{r\varepsilon m}},{ v_{r\varepsilon m}})}\nabla {c_{r\varepsilon m}}\cdot \nabla \varphi \, dx \nonumber \\&\qquad + \int _{\varOmega }{ {\bar{c}}_{r\varepsilon m}\chi ({\bar{c}}_{r\varepsilon m},v_{r\varepsilon m})} { G_{\varepsilon }({{{\mathcal {R}}}}_{r}(\partial _cg({\bar{c}}_{r\varepsilon m},v_{r\varepsilon m})\nabla c_{r\varepsilon m}))\cdot \nabla \varphi )}\,dx\nonumber \\&\qquad +\int _{\varOmega }{ {\bar{c}}_{r\varepsilon m}\chi ({\bar{c}}_{r\varepsilon m},v_{r\varepsilon m})}{ G_{\varepsilon }({{{\mathcal {R}}}}_{r} (\partial _vg({\bar{c}}_{r\varepsilon m},v_{r\varepsilon m})\nabla v_{r\varepsilon m})) \cdot \nabla \varphi }\nonumber \\&\qquad +f_c({ {\bar{c}}_{r\varepsilon m},v_{r\varepsilon m}})\varphi \, dx, \end{aligned}$$

5.26b $$\begin{aligned}&c_{{r\varepsilon m}}(0,\cdot )=c_0\quad \text {in }L^2(\varOmega ) \end{aligned}$$

and

5.26c $$\begin{aligned}&{\partial _t v_{r\varepsilon m}=D_v\varDelta v_{r\varepsilon m}+f_v( {\bar{c}}_{r\varepsilon m},v_{r\varepsilon m})}\quad&{ \text {a.e. in }(0,T)\times \varOmega }, \end{aligned}$$

5.26d $$\begin{aligned}&{ D_v \partial _{\nu } v_{r\varepsilon m}=0\quad }&{\text {a.e. in }(0,T)\times \partial \varOmega }, \end{aligned}$$

5.26e $$\begin{aligned}&{v_{r\varepsilon m}}(0,\cdot )=v_0\quad&\text {in }H^1(\varOmega ). \end{aligned}$$

From (5.12) and (5.21) we conclude that the sequence $\{ { v_{r\varepsilon m}} \}$ is uniformly bounded in $L^2(0,T;H^{2}(\varOmega ))$ and $\partial _t v_{r\varepsilon m}\in L^2(0,T;(L^2(\varOmega ))$. Hence the Lions-Aubin lemma and the Banach–Alaoglu theorem imply that there exists $v_{r\varepsilon }$ s.t. (after switching to a subsequence, if necessary)

5.27 $$\begin{aligned} {v_{r\varepsilon m}}&\underset{m\rightarrow \infty }{\rightharpoonup } {v_{r\varepsilon }} \quad \text {in }L^2(0,T;{ H^2}(\varOmega )),\nonumber \\ \partial _t {v_{r\varepsilon m}}&\underset{m\rightarrow \infty }{\rightharpoonup } \partial _t{ v_{r\varepsilon }}\quad \text {in }L^2(0,T;{ L^2(\varOmega )}),\nonumber \\ {v_{r\varepsilon m}}&\underset{m\rightarrow \infty }{\rightarrow }{ v_{r\varepsilon }} \quad \text {in }L^2(0,T;{ H^1}(\varOmega )) {\text { and a.e. in } (0,T) \times \varOmega }, \end{aligned}$$

and this ${v_{r\varepsilon }}$ satisfies equation (5.11c) for ${\bar{c}}_{r\varepsilon }$ as well as the initial and boundary conditions in the required sense.

Further, due to (5.24), and (5.25) we have in the usual way that

5.28 $$\begin{aligned} c_{r\varepsilon m}(t,\cdot ) \underset{m \rightarrow \infty }{\rightharpoonup } c_{r\varepsilon }(t,\cdot )\quad \text {in } L^2(\varOmega )\quad \text { for all }t>0. \end{aligned}$$

In particular,

$$\begin{aligned} c_{r\varepsilon m}(0,\cdot )=c_0, \end{aligned}$$

i.e. the initial condition is satisfied.

It remains now to pass to the limit in (5.26a). For this purpose we use the Minty–Browder method. To shorten the notation, we introduce for $m \in {\mathbb {N}}\cup \{\infty \}$

$$\begin{aligned}&\langle {{{\mathcal {M}}}}_m(u), \varphi \rangle _{{ L^2(0,T;(H^1(\varOmega ))^*),L^2(0,T;H^1(\varOmega ))}}\\&\quad := \int _0^T\int _{\varOmega } D_c({\bar{c}}_{r\varepsilon m}, v_{r\varepsilon m}) \nabla u\cdot \nabla \varphi \\&\qquad - G_{\varepsilon }({{{\mathcal {R}}}}_r(\partial _c g ({\bar{c}}_{r\varepsilon m}, v_{r\varepsilon m}) \nabla u)) {\bar{c}}_{r\varepsilon m} \chi ({\bar{c}}_{r\varepsilon m}, v_{r\varepsilon m})\cdot \nabla \varphi \, dxdt,\\&\qquad \langle f_m, \varphi \rangle _{{ L^2(0,T;(H^1(\varOmega ))^*),L^2(0,T;H^1(\varOmega ))}}\\&\quad :=\int _0^T\int _{\varOmega }{{\bar{c}}_{r\varepsilon }\chi ({\bar{c}}_{r\varepsilon m},v_{r\varepsilon m})}{G_{\varepsilon }({{{\mathcal {R}}}}_{r} (\partial _vg({\bar{c}}_{r\varepsilon m},v_{r\varepsilon m})\nabla v_{r\varepsilon m})) \cdot \nabla \psi }\\&\qquad +f_c({ {\bar{c}}_{r\varepsilon m},v_{r\varepsilon m}})\psi \, dxdt, \end{aligned}$$

where

$$\begin{aligned} {\bar{c}}_{r\varepsilon \infty } := {\bar{c}}_{r\varepsilon },\quad {\bar{v}}_{r\varepsilon \infty } := {\bar{v}}_{r\varepsilon }. \end{aligned}$$

Due to Lemma 4.2(ii) and (5.14) each operator ${{{\mathcal {M}}}}_m$ is monotone, hemicontinuous, and satisfiesConsequently, there is $\eta \in L^2(0,T;(H^1(\varOmega ))^*)$ s.t.

5.29 $$\begin{aligned} {{{\mathcal {M}}}}_m(c_{r\varepsilon m}) \rightharpoonup \eta \text { in } L^2(0,T;(H^1(\varOmega ))^*). \end{aligned}$$

Next, from (5.23) and (5.27) we conclude using the boundedness and continuity of functions $G_{\varepsilon },\nabla g,\nabla f_c$, and $(c,v)\mapsto c\chi (c,v)$ over $\mathbb {R}\times \mathbb {R}^+_0$ and of operator ${{{\mathcal {R}}}}_{r}$ in $L^2(\varOmega )$ and the dominated convergence theorem that

5.30 $$\begin{aligned} f_m\underset{m \rightarrow \infty }{\rightarrow }f_{\infty }\quad \text {in }L^2(0,T;(H^1(\varOmega ))^*). \end{aligned}$$

A similar argument yields

$$\begin{aligned} {{{\mathcal {M}}}}_m(w)\underset{m \rightarrow \infty }{\rightarrow }{{{\mathcal {M}}}}_{\infty }(w),\quad \text {in }L^2(0,T;(H^1(\varOmega ))^*) \end{aligned}$$

so that due to (5.24) and the compensated compactness

$$\begin{aligned}&\langle {{{\mathcal {M}}}}_m(w),c_{r\varepsilon m}\rangle _{{ L^2(0,T;(H^1(\varOmega ))^*),L^2(0,T;H^1(\varOmega ))}}\\ \underset{m \rightarrow \infty }{\rightarrow }&\langle {{{\mathcal {M}}}}_{\infty }(w),c_{r\varepsilon }\rangle _{{ L^2(0,T;(H^1(\varOmega ))^*),L^2(0,T;H^1(\varOmega ))}}. \end{aligned}$$

Observe that the weak formulation (5.26a) is equivalent to

5.31 $$\begin{aligned} \partial _t c_{r\varepsilon m}= -{{{\mathcal {M}}}}_m(c_{r\varepsilon m})+f_m\quad \text {in }(H^1(\varOmega ))^*. \end{aligned}$$

Combining (5.25), (5.29), and (5.30) we can pass to the weak limit in (5.31) and obtain

5.32 $$\begin{aligned} \partial _t c_{r\varepsilon }= -\eta +f_{\infty }\quad \text {in }(H^1(\varOmega ))^*. \end{aligned}$$

For $w \in L^2(0,T;H^1(\varOmega ))$ and $m\in {\mathbb {N}}$ we have due to the monotonicity of ${{{\mathcal {M}}}}_m$ that

5.33 $$\begin{aligned} X_m := \langle {{{\mathcal {M}}}}_m (c_{r\varepsilon m})-{{{\mathcal {M}}}}_m (w), c_{r\varepsilon m}-w\rangle _{{(H^1(\varOmega ))^*,H^1(\varOmega )}}\ge 0. \end{aligned}$$

Moreover, setting $\varphi = c_{r\varepsilon m}$ in (5.26) and inserting the obtained term into the definition of $X_m$, we conclude that

5.34 $$\begin{aligned} X_m&= - \langle {{{\mathcal {M}}}}_m ( c_{r\varepsilon m}), w\rangle _{{ L^2(0,T;(H^1(\varOmega ))^*),L^2(0,T;H^1(\varOmega ))}}\nonumber \\&\quad - \langle {{{\mathcal {M}}}}_m ( w), c_{r\varepsilon m}-w\rangle _{{ L^2(0,T;(H^1(\varOmega ))^*),L^2(0,T;H^1(\varOmega ))}}\nonumber \\&\quad + \frac{1}{2}\Vert c_0 \Vert _{L^2(\varOmega )}^2-\frac{1}{2}\Vert c_{r\varepsilon m}(T) \Vert _{L^2(\varOmega )}^2\nonumber \\&\quad +\langle f_m, c_{r\varepsilon m}\rangle _{{ L^2(0,T;(H^1(\varOmega ))^*),L^2(0,T;H^1(\varOmega ))}}. \end{aligned}$$

Combining (5.28) for $t=T$, (5.29), (5.33), (5.34), (5.22), and (5.24), we obtain

$$\begin{aligned} 0&\le \limsup _{m \rightarrow \infty } X_m\\&\le - \langle \eta , w\rangle _{{ L^2(0,T;(H^1(\varOmega ))^*),L^2(0,T;H^1(\varOmega ))}}\\&\quad - \langle {{{\mathcal {M}}}}_{\infty } (w), c_{r\varepsilon }-w\rangle _{{ L^2(0,T;(H^1(\varOmega ))^*),L^2(0,T;H^1(\varOmega ))}}\\&\quad + \frac{1}{2}\Vert c_0 \Vert _{L^2(\varOmega )}^2-\frac{1}{2}\Vert c_{r\varepsilon }(T) \Vert _{L^2(\varOmega )}^2+\langle f_{\infty }, c_{r\varepsilon }\rangle _{{ L^2(0,T;(H^1(\varOmega ))^*),L^2(0,T;H^1(\varOmega ))}}. \end{aligned}$$

As $c_{r\varepsilon }$ satisfies (5.32), it follows from the last inequality that

$$\begin{aligned} 0 \le \langle \eta -{{{\mathcal {M}}}}_{\infty }(w), c_{r\varepsilon }-w \rangle _{{ L^2(0,T;(H^1(\varOmega ))^*),L^2(0,T;H^1(\varOmega ))}} \end{aligned}$$

holds for all $w \in L^2(0,T;H^1(\varOmega ))$.

Since ${{{\mathcal {M}}}}_{\infty }$ is monotone and hemicontinuous, Minty’s lemma implies that it is maximal monotone. Consequently, $\eta ={{{\mathcal {M}}}}_{\infty }(c_{r\varepsilon })$.

Altogether, we conclude that $(c_{r\varepsilon },v_{r\varepsilon })$ satisfies (5.11) for ${\bar{c}}_{r\varepsilon }$, meaning that $\varPhi ( {\bar{c}}_{r\varepsilon }) = c_{r\varepsilon }$ holds, i.e. $\varPhi$ is a closed operator. Together with (5.20), this implies that

5.35 $$\begin{aligned} \varPhi : L^2(0,T; L^2(\varOmega )) \rightarrow L^2(0,T; L^2(\varOmega )) \text { is a compact operator.} \end{aligned}$$

Since we aim to apply the Leray–Schauder principle (Zeidler 1986, Chapter 6, §6.8, Theorem 6.A), it is necessary to consider for $\lambda \in (0,1)$ the system which corresponds to $c_r=\lambda \varPhi (c_r)$. The corresponding weak-strong formulation reads:

5.36a $$\begin{aligned}&\langle \partial _t c_{r\varepsilon },\varphi \rangle _{{(H^1(\varOmega ))^*,H^1(\varOmega )}}\nonumber \\&\quad =-\int _{\varOmega }D_c( c_{r\varepsilon },v_{r\varepsilon })\nabla c_{r\varepsilon }\cdot \nabla \varphi \, dx\nonumber \\&\qquad + \int _{\varOmega } c_{r\varepsilon }\chi ( c_{r\varepsilon },v_{r\varepsilon })\lambda G_{\varepsilon }(\lambda ^{-1}{{{\mathcal {R}}}}_{r}(\partial _cg( c_{r\varepsilon },v_{r\varepsilon })\nabla c_{r\varepsilon }))\cdot \nabla \varphi \,dx\nonumber \\&\qquad +\lambda \int _{\varOmega }G_{\varepsilon }({{{\mathcal {R}}}}_{r} (\partial _vg( c_{r\varepsilon },v_{r\varepsilon })\nabla v_{r\varepsilon })) \cdot c_{r\varepsilon }\chi ( c_{r\varepsilon },v_{r\varepsilon })\nabla \varphi +f_c( c_{r\varepsilon },v_{r\varepsilon })\varphi \,\ dx,\end{aligned}$$

5.36b $$\begin{aligned}&c_{{r\varepsilon }}(0,\cdot )=\lambda c_0\quad \text {in }L^2(\varOmega ) \end{aligned}$$

and

5.36c $$\begin{aligned}&{\partial _t v_{r\varepsilon }=D_v\varDelta v_{r\varepsilon }+f_v( c_{r\varepsilon },v_{r\varepsilon })}\quad&{ \text {a.e. in }(0,T)\times \varOmega }, \end{aligned}$$

5.36d $$\begin{aligned}&{D_v \partial _{\nu } v_{r\varepsilon }=0} \quad&{\text {a.e. in }(0,T)\times \partial \varOmega }, \end{aligned}$$

5.36e $$\begin{aligned}&{v_{r\varepsilon }}(0,\cdot )=v_0\quad&\text {in }H^1(\varOmega ). \end{aligned}$$

Taking $\varphi := c_{r\varepsilon }$ in (5.36) and estimating the right-hand side by using Assumptions 5.1, and 5.4(a), the Hölder inequality, and the fact that $|G_{\varepsilon }(x)| \le |x|$, we obtain thatholds for a.e. $t\in (0,T)$. Further, performing estimates similar to the proof of Theorem 5.13 below and using (5.12), we conclude that the set

$$\begin{aligned} \left\{ c_r \in L^2(0,T;L^2(\varOmega )): \, c_r = \lambda \varPhi (c_r) \text { for } \lambda \in (0,1)\right\} \end{aligned}$$

is uniformly bounded. Consequently, for all $\varepsilon \in (0,1)$ the Leray-Schauder principle implies that $\varPhi$ has a fixed point $c_{r\varepsilon }$, which together with the corresponding $v_{r\varepsilon }$, satisfies (5.9) in the weak-strong sense on the interval [0, *T*]. Since $T>0$ was arbitrary, the standard prolongation argument yields the existence of a global solution.

It remains to check that $c_{r\varepsilon }$ is nonnegative. Taking $\varphi :=-(c_{r\varepsilon })_- =\min \{c_{r\varepsilon },0\}$ in (5.10) and using $f_c(0,\cdot )\equiv 0$, the boundedness of $G_{\varepsilon },D_c,\partial _c f_c$, and $(c,v)\mapsto c\chi (c,v)$, along with the Hölder and Young inequalities, yieldsSince $c_{r\varepsilon }(0,\cdot )=c_0 \ge 0$, the Gronwall inequality implies that $(c_{r\varepsilon })_{-} = 0$, i.e. that $c_{r\varepsilon }\ge 0$. $\square$

#### Remark 5.12

Observe that $c_{r\varepsilon }$ cannot be replaced by $-(c_{r\varepsilon })_-$ inside the nonlocal operator. This is why we introduced the flux-limitation.

Now we are ready to prove Theorem 5.10.

#### Proof of Theorem 5.10

We start with the case

$$\begin{aligned} D_v >0. \end{aligned}$$

Lemma 5.11 gives the existence of solutions $(c_{r\varepsilon }, v_{r\varepsilon })$ to (5.9). Setting $\varphi = c_{r\varepsilon }$ in (5.10), using the facts that $f_c$ is Lipschitz and $|G_{\varepsilon }(x)| \le |x|$, we can estimate similarly to Theorem 5.13 below and obtain upper bounds of the form (5.41)–(5.47), which are independent from $\varepsilon$ (with $p=q=2$ there). Applying the Lions-Aubin lemma and the Banach–Alaoglu theorem, we conclude the existence of a pair of nonnegative functions $c_r$ and $v_r$ having the regularity stated in Definition 5.6 and such that for a sequence $\varepsilon _m \underset{m \rightarrow \infty }{\rightarrow } 0$ it holds that

5.37 $$\begin{aligned} c_{r\varepsilon _m}&\underset{m \rightarrow \infty }{\rightarrow } c_r \text { in } L^2(0,T;L^2(\varOmega )) \text { and a.e. in } (0,T) \times \varOmega , \end{aligned}$$

5.38 $$\begin{aligned} v_{r\varepsilon _m}&\underset{m \rightarrow \infty }{\rightarrow } v_r \text { in } L^2(0,T;H^1(\varOmega )) \text { and a.e. in } (0,T) \times \varOmega , \end{aligned}$$

5.39 $$\begin{aligned} c_{r\varepsilon _m}&\underset{m \rightarrow \infty }{\rightharpoonup } c_r \text { in } L^2(0,T;H^1(\varOmega )). \end{aligned}$$

Consider an arbitrary measurable set $E \subset (0,T) \times \varOmega$. Using $G_{\varepsilon }(x)-x = -\varepsilon \frac{x|x|}{1+\varepsilon |x|}$, we can estimate for every component $i \in \{1,\ldots ,n\}$:where the last term tends to 0 as $\varepsilon _m \underset{m \rightarrow \infty }{\rightarrow } 0$. As the term inside the integral is moreover bounded in $L^2(0,T;L^2(\varOmega ))$ by a constant independent from $\varepsilon _m$, we conclude by using a result from Evans (1990, p. 6) that in $L^2(0,T;(L^2(\varOmega ))^n)$

$$\begin{aligned} { G}_{\varepsilon _m}({{{\mathcal {R}}}}_{r}(\partial _cg(c_{r\varepsilon _m},v_{r\varepsilon _m})\nabla c_{r\varepsilon _m}))-{{{\mathcal {R}}}}_{r}(\partial _cg(c_{r\varepsilon _m},v_{r\varepsilon _m})\nabla c_{r\varepsilon _m}) \underset{m \rightarrow \infty }{\rightharpoonup } 0. \end{aligned}$$

From this and the boundedness of $\Vert \nabla c_{r\varepsilon _m}\Vert _{L^2(0,T;(L^2(\varOmega ))^n)}$, (5.37)–(5.39), Lemmas 3.5 or 3.7(i) and (ii), respectively, the fact that $|G_{\varepsilon }(x)| \le |x|$, the continuity of $\partial _c g, \chi$, (5.3), (5.4), compensated compactness, the dominated convergence theorem, and the Hölder inequality, we obtain that for all $\psi \in L^2(0,T;H^1(\varOmega ))$ it holds that

$$\begin{aligned}&\int _0^T \int _{\varOmega } { G}_{\varepsilon _m}({{{\mathcal {R}}}}_{r}(\partial _cg(c_{r\varepsilon _m},v_{r\varepsilon _m})\nabla c_{r\varepsilon _m}))\cdot c_{r\varepsilon _m}\chi (c_{r\varepsilon _m},v_{r\varepsilon _m}) \nabla \psi \,dx \, dt\\ \underset{m \rightarrow \infty }{\rightarrow }&\int _0^T \int _{\varOmega } {{{\mathcal {R}}}}_{r}(\partial _cg(c_r,v_r)\nabla c_r)\cdot c_r\chi (c_r,v_r) \nabla \psi \,dx \, dt. \end{aligned}$$

The convergence to the remaining terms in (5.8a) and the rest of (5.8) can be obtained in a way either completely analogous or very similar to the corresponding parts of the proof of Lemma 5.11.

In order to prove existence for the case

$$\begin{aligned} D_v = 0 \end{aligned}$$

consider a family of solutions $(c_{rD_v}, v_{rD_v})$ corresponding to $D_v \in (0,1)$. Estimating similarly to the proof of Theorem 5.13 below and performing a standard limit procedure based on the Banach–Alaoglu theorem, the dominated convergence theorem, the Lions lemma (Lions 1969, Lemma 1.3), and the compensated compactness, one readily obtains a solution $(c_{r0},v_{r0})$ for $D_v = 0$ in the sense of Definition 5.6. Observe that this time the gradient of *v*-component enters linearly, so that no strong convergence is required. We omit further details. $\square$

### Global existence of solutions to (5.1): the case of $f_c$ dissipative

In this subsection we provide an extension of the existence Theorem 5.10 from Sect. 5.2:

#### Theorem 5.13

Let Assumptions 1.1, 5.1, and 5.3(b) hold and let *r* satisfy Assumptions 5.4(a). Set^2^

5.40 $$\begin{aligned} q := \min \left\{ 2, \frac{s+1}{s}\right\} ,\quad q^*:=\frac{q}{q-1}. \end{aligned}$$

Then there exists a global weak-strong solution to (5.1) in terms of Definition 5.6, with $\partial _t c_r\in L^q(0,T;(W^{1,q^*}(\varOmega ))^*)$ and satisfying the following estimates: For all $T>0$5.415.425.435.445.455.465.475.48

#### Proof

For $$k \in {\mathbb {N}}$$ set

$$\begin{aligned} f_{ck}(c,v) := f_c(c, v)\eta _k(c), \end{aligned}$$

where $\eta _k$ is a cut-off function:

5.49 $$\begin{aligned} \eta _k \in C_0^{\infty }(B_k(0)) \quad \text {with}\quad \eta _k \equiv 1 \quad \text { in } B_{k-1}(0) \quad \text {and}\quad 0 \le \eta _k \le 1. \end{aligned}$$

Since $f_{ck}$ is Lipschitz, Theorem 5.10 implies the existence of a solution $(c_{rk},v_{rk})$ in terms of Definition 5.6 with $\partial _t c_{rk}\in L^2(0,T;(H^1(\varOmega ))^*)$, which corresponds to $f_c=f_{{ c}k}$. Our next aim is to prove that $(c_{rk},v_{rk})$ satisfies the same bounds as in the statement of the Theorem with some constant  which does not depend upon *k*.

SetTaking $\varphi :=c_{rk}$ in (5.8a) written for $c_{rk}$ and using Assumptions 5.1, 5.3(b), 5.4(a) and the Hölder and Young inequalities, we compute5.50Next, we estimate $v_{rk}$. If $D_v>0$, then standard theory (Ladyzhenskaya et al. 1968) yields that for all $0<t\le T$5.51Here and further in the proof we omit the dependence of constants upon $D_v$. If $D_v=0$, then we get the ODE

5.52 $$\begin{aligned} \partial _t v_{rk}=&f_v(c_{rk},v_{rk}). \end{aligned}$$

Hence, the assumptions on $f_v$ and the solution components together with the chain rule imply that

$$\begin{aligned} \partial _t v_{rk}\in&L^2(0,T;H^1(\varOmega )). \end{aligned}$$

Computing the gradient on both sides of (5.52), multiplying by $\nabla v_{rk}$ throughout, integrating over $\varOmega$, and using Assumptions 5.1 and the Young inequality, we obtain that5.53Applying the Gronwall inequality to (5.53) yields5.54Multiplying (5.52) by $v_{rk}$ we obtain in a similar fashion that5.55Adding (5.54) and (5.55) together yields5.56Estimating the right-hand side of (5.52) by using (5.55) implies5.57Further, combining (5.50) with (5.51) if $D_v>0$ and with (5.56) if $D_v=0$ and using the Gronwall inequality yields for $c_{rk}$ the same estimates as (5.41) and (5.42), and the estimate5.58From (5.6) and (5.58), the embedding of Lebesgue spaces, and $\eta _k \in [0,1]$ we conclude thatso that (5.47) holds for $f_{ck}(c_{rk},v_{rk})$. Combining (5.41) and (5.42) for $c_{rk}$ with (5.51) or (5.56) and (5.57) (depending on the sign of $D_v$) and using the equation for $v_{rk}$ yields such bounds as (5.44)–(5.46) and (5.48) for $c_{rk}$ and $v_{rk}$. Finally, combining Assumptions 5.1 with bounds on $\nabla c_{rk},\nabla v_{rk}$, and $f_{ck}(c_{rk},v_{rk})$, the weak formulation (5.8a), and estimating in a standard way yields (5.43) for $\partial _t c_{rk}$.

Since $(c_{rk},v_{rk})$ satisfy (5.41)–(5.48) uniformly in *k*, a standard limit procedure based on the Banach–Alaoglu theorem, the dominated convergence theorem, the Lions lemma, and the compensated compactness yields the existence of a weak-strong solution $(c_{r},v_{r})$ to (5.8) which satisfies (5.41)–(5.48). $\square$

### Limiting behaviour of the nonlocal model (5.1) as $r\rightarrow 0$

In this subsection we finally prove our main result concerning convergence for $r\rightarrow 0$.

#### Proof of Theorem 5.8

Due to (5.7) and Lemma 3.5 (3.5) or 3.7 (3.5), respectively, there exists a sequence $r_m\rightarrow 0$ as $m\rightarrow \infty$ such thatSince for each such $r_m$ the Assumptions 5.4(a) are satisfied, Theorem 5.13 is applicable and yields the existence of solutions $(c_{r_m},v_{r_m})$ which satisfy (5.41)–(5.48). Replacing $\Vert {\mathcal {R}}_r \Vert$ by  in  makes the constant in (5.41)–(5.48) independent of *m*. Using the Lions-Aubin lemma and the Banach–Alaoglu theorem we conclude (by possibly switching to a subsequence) that

5.59 $$\begin{aligned}&c_{r_m} \underset{m \rightarrow \infty }{\rightarrow } c,\ \ v_{r_m} \underset{m \rightarrow \infty }{\rightarrow } v \qquad&\text {in } L^2(0,T;L^2(\varOmega )), \quad \text {a.e. in } (0,T)\times \varOmega \end{aligned}$$

5.60 $$\begin{aligned}&c_{r_m} \underset{m \rightarrow \infty }{\rightharpoonup } c,\ \ v_{r_m} \underset{m \rightarrow \infty }{\rightharpoonup } v \qquad&\text {in } L^2(0,T;H^1(\varOmega )). \end{aligned}$$

Using standard arguments based on the Banach–Alaoglu theorem, the dominated convergence theorem, the Lions lemma, and assumptions on $\chi$ and *g* we conclude from (5.59) and (5.60) that

5.61 $$\begin{aligned}&c_{r_m}\chi (c_{r_m}, v_{r_m})\underset{m \rightarrow \infty }{\rightarrow } c\chi (c,v) \qquad&\text {in } L^2(0,T;L^2(\varOmega )), \end{aligned}$$

5.62 $$\begin{aligned}&g(c_{r_m},v_{r_m}) \underset{m \rightarrow \infty }{\rightharpoonup } g(c,v) \qquad&\text {in } L^2(0,T;H^1(\varOmega )). \end{aligned}$$

Observe that for any $\psi \in L^{\infty }(0,T; W^{1, \infty }(\varOmega ))$ the following estimate holds:

5.63 $$\begin{aligned}&\int _0^T \int _{\varOmega } \left| {\mathcal {R}}_{r_m} (c_{r_m}\chi (c_{r_m}, v_{r_m}) \nabla \psi )-c \chi (c,v) \nabla \psi \right| ^2 \, dx \, dt \end{aligned}$$

5.64 $$\begin{aligned}&\quad \le 2\left( \int _0^T \int _{\varOmega } \left| {\mathcal {R}}_{r_m} (c_{r_m}\chi (c_{r_m}, v_{r_m}) \nabla \psi )-{\mathcal {R}}_{r_m} (c\chi (c, v) \nabla \psi ) \right| ^2 \, dx \, dt\right. \nonumber \\&\qquad \left. + \int _0^T \int _{\varOmega } \left| {\mathcal {R}}_{r_m} (c\chi (c, v) \nabla \psi )-c \chi (c,v) \nabla \psi \right| ^2 \, dx \, dt \right) . \end{aligned}$$

Now, using (5.61) together with Lemma 3.5(i) and (iii) and (3.6) or (3.7)(i) and (iii) and (3.9), respectively, we conclude that the right hand side of (5.64) tends to zero, hence

5.65 $$\begin{aligned} {{{\mathcal {R}}}}_{r_m} (c_{r_m}\chi (c_{r_m},v_{r_m})\nabla \psi ) \underset{m \rightarrow \infty }{\rightarrow } c\chi (c,v)\nabla \psi \quad \text {in } L^2(0,T;(L^2(\varOmega ))^n). \end{aligned}$$

Thus, using Lemma 3.5(ii) or Lemma 3.7(ii), respectively, and compensated compactness, we obtain from (5.62) and (5.65) that

$$\begin{aligned}&\int _0^T\int _{\varOmega }c_{r_m}\chi (c_{r_m},v_{r_m}) {{{\mathcal {R}}}}_{r_m}(\nabla g(c_{r_m},v_{r_m}))\cdot \nabla \psi \,dx\, dt \\&\quad =\int _0^T\int _{\varOmega } \nabla g(c_{r_m},v_{r_m})\cdot {{{\mathcal {R}}}}_{r_m}(c_{r_m}\chi (c_{r_m},v_{r_m})\nabla \psi )\,dxdt \\ \underset{m \rightarrow \infty }{\rightarrow }&\int _0^T\int _{\varOmega } \nabla g(c,v)\cdot c\chi (c,v)\nabla \psi \,dxdt. \end{aligned}$$

The convergence in the remaining terms, equations, and conditions follows by means of a standard limit procedure based on the Banach–Alaoglu theorem, the dominated convergence theorem, the Lions lemma, and the compensated compactness. We omit these details. $\square$

## Numerical simulations in 1D

We perform numerical simulations to investigate on the one hand the effect of differences between hitherto choices of nonlocal operators and our novel ones proposed in Sect. 3, and on the other hand convergence between nonlocal and local formulations. For compactness, our current study restricts to the prototypical nonlocal model for cellular adhesion (1.1), its reformulation as (5.1), and the corresponding local model (5.2). Thus, for (5.1) we take the operator form ${\mathcal {R}}_r={\mathcal {T}}_r$, with ${\mathcal {T}}_r$ as in (3.2). These models can be interpreted in the context of a population of cells invading an adhesion-laden ECM/tissue environment and, with this in mind, we initially concentrate cells at the centre of a one-dimensional domain $\varOmega = [0,L]$ and impose an initially homogeneous ECM. Specifically, we set for the ECM

6.1 $$\begin{aligned} v_0(x) = 1,\quad x \in \varOmega \end{aligned}$$

and consider for the cell population a Gaussian-shaped aggregate

6.2 $$\begin{aligned} c_0(x)=\exp \left( -\alpha (x-x_c)^2 \right) ,\quad x \in \varOmega , \end{aligned}$$

where we set $x_c = L/2$ or $x_c = 0$.

The numerical scheme follows that described in Gerisch (2010), which we refer to for details. Briefly, a Method of Lines approach is invoked whereby equations are first discretised in space (in conservative form, via a finite volume method) to yield a high-dimensional system of ODEs, which are subsequently integrated in time. Discretisation of advective terms follows a third order upwinding scheme, augmented by flux limiting to preserve positivity of solutions and the resulting scheme is (approximately) second-order accurate in space. Time integration has been performed with standard Matlab ODE solvers: our default is “ode45” with absolute and relative error tolerances set at $10^{-6}$, but simulations have been compared for varying space discretisation step, ODE solver, and error tolerances. To measure the difference between two distinct solutions over time we define a distance function as follows:where $u_1$ and $u_2$ denote the two solutions that are being compared.

### Comparison of nonlocal operator representations

We first explore the correspondence between forms of nonlocal operator representation: we choose the prototypical nonlocal model for cell/matrix adhesion (1.1) and its reformulation (5.1), therefore taking for the latter the operator form ${\mathcal {R}}_r={\mathcal {T}}_r$ with ${\mathcal {T}}_r$ as in (3.2). In what follows, solutions to (1.1) are denoted $c_{A}$ and $v_{A}$ and those for (5.1) denoted $c_{T}$ and $v_{T}$. For simplicity we restrict in this section to a minimalist formulation in which $D_c =$ constant, $\chi = 1$, $f_c = 0$. Cell–matrix interactions are defined by $g(c,v) = S_{cc} c + S_{cv} v$ and $f_v(c,v) = -\mu c v$, where $S_{cc}$ and $S_{cv}$ respectively represent cell-to-cell and cell-to-matrix adhesion strengths and $f_v$ simplistically describes (direct) proteolytic degradation of matrix by cells parametrised by degradation rate $\mu$.

**Fig. 1:**
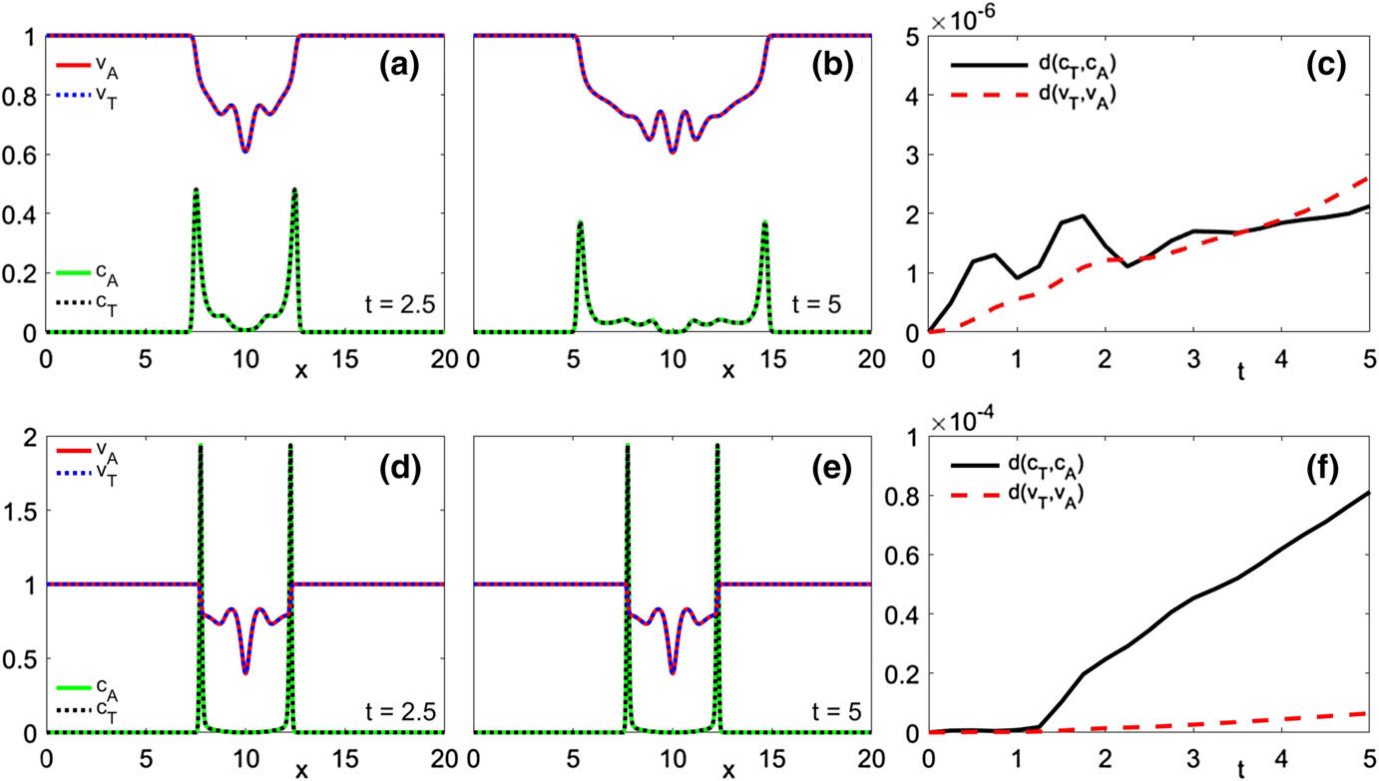
Comparison between nonlocal formulations (1.1) and (5.1). **a**–**b** Cell and matrix densities for the models (1.1) and (5.1) at $t=2.5$ and $t = 5$. **c** Difference between the solutions. For these simulations we take $\alpha = 10$, $r = 1$, $D_c = 0.01$, $\chi = 1$, $F_r = 2$, $f_c = 0$ and $f_v(c,v) = - c v$, along with **a**–**c** $g(c,v) = 10 v$, **d**–**f** $g(c,v) = 2.5c + 10 v$

Figure 1 shows the computed solutions under (a–c) negligible cell–cell adhesion ($S_{cc} = 0$) and (d-f) moderate cell–cell adhesion ($S_{cc} = S_{cv}/4$). The equivalence of the two formulations is revealed through the negligible difference between solutions, with the distance magnitude attributable to the subtly distinct numerical implementation. Both simulations describe an invasion/infiltration process, in which matrix degradation by the cells generates an adhesive gradient that pulls cells into the acellular surroundings. The impact of cell–cell adhesion is manifested in the compaction of cells at the leading edge into a tight aggregate.

**Fig. 2:**
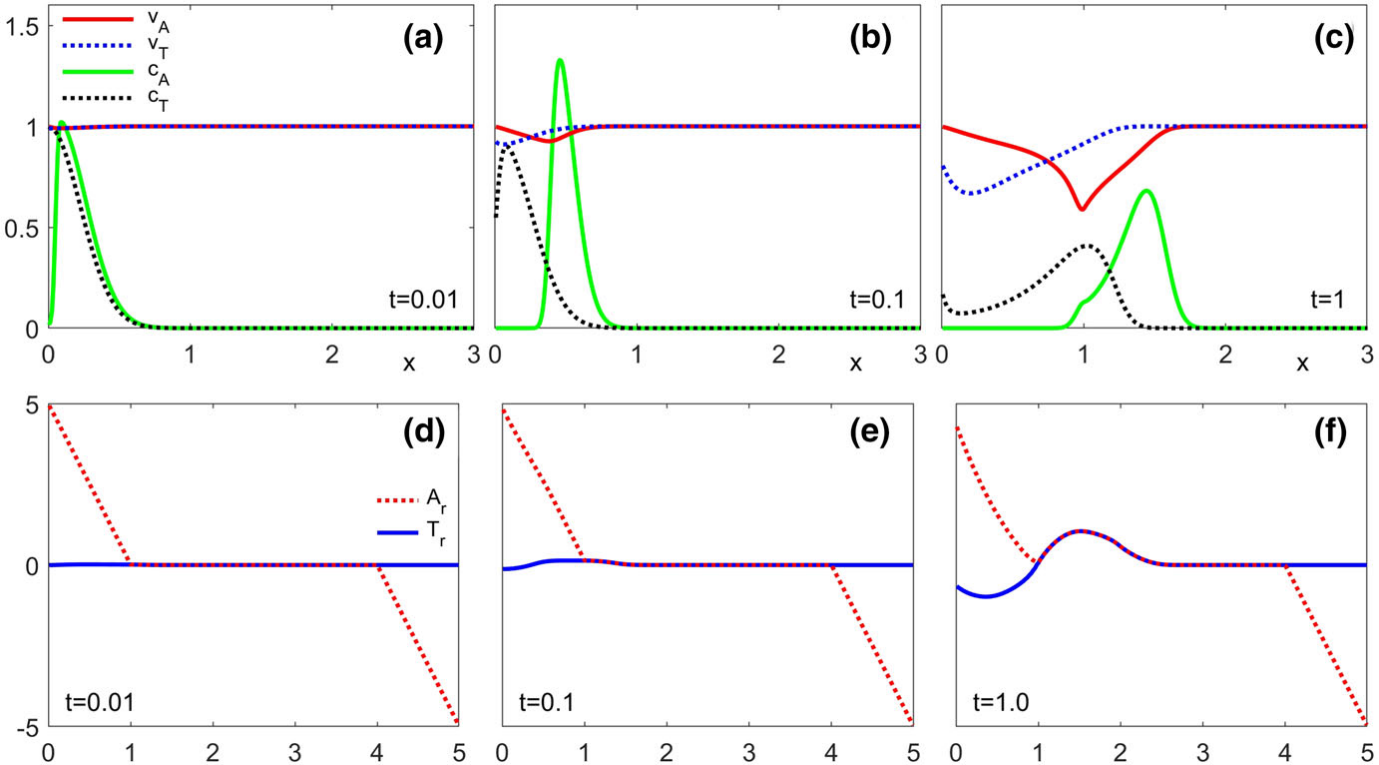
**a**–**c** Comparison between nonlocal formulations (1.1) and (5.1) near boundaries. Model as in Fig. 1a–c, but with the cells initially concentrated at the boundary. **d**, **f** Comparison of the two forms of nonlocal operator corresponding to the simulations represented in (**a**–**c**). The operators are practically identical sufficiently far from the boundary, but can diverge significantly for distances $<r$ from the boundaries

However, as pointed out in Sect. 3, differences in the nonlocal formulations can emerge in the vicinity of boundaries. To highlight this we consider an equivalent formulation to Fig. 1a–c, but with the cells initially placed at the left boundary [$x_{c} = 0$ in (6.2)], e.g. suggesting a tumor mass which is concentrated there and whose cells are expected to detach and migrate into the considered 1D domain, travelling from left to right. As stated earlier we impose zero-flux boundary conditions at $x=0$ (and $x=L$), and further suppose $c = v = 0$ and $\nabla c = \nabla v$ in the extradomain region ($\mathbb {R}\backslash \varOmega$). Representative simulations are shown in Fig. 2. They are in agreement with our observation in Example 3.3. Indeed, for this scenario, in the prototypical nonlocal model (1.1)–(1.2) there is a very large adhesion velocity modulus at $x=0$; the cells are crowded within the tumor mass and their mutual interactions are maintained during the invasion process in a sufficiently strong manner to ensure a collective shift of the still concentrated cell aggregate, with a correspondingly strong tissue degradation in its wake. In the reformulation (5.1)–(3.2), rather, the adhesion magnitude at $x=0$ is for the same initial condition much lower - suggesting a tumor whose cells are readier to detach and migrate individually. This results in a more diffusive spread, with accordingly less degradation of tissue, and with cell mass remaining available at the original site over a larger time span. The latter scenario is different from the former one, but it seems nevertheless reasonable, as a tumor mass would very often not move as a whole from its original location to another in a relatively short time; moreover, the active cells in a sufficiently large tumor (releasing substantial amounts of acidity) are known to preferentially adopt a migratory phenotype and perform EMT (epithelial-mesenchymal transition), see e.g., Gupta (2015), Peppicelli et al. (2014) and Prieto-García et al. (2017), which supports the idea of cells moving in a loose way rather than in compact, highly aggregated assemblies.^3^ As such, our simulations suggest that, within this particular function- and parameter setting, choosing the adhesion operator in the form (1.2) instead of (3.2) might possibly overestimate the tumor invasion speed and associated healthy tissue degradation, thereby predicting a spatially concentrated tumor and neglecting regions with lower cell densities which can nevertheless trigger tumor recurrence if untreated.

### Comparison between nonlocal and local formulation

Having compared together the original, (1.1), and the new, (5.1), nonlocal formulations, we next consider the extent to which their dynamics can be captured by the classical local formulation (5.2). Note that for nonlocal model simulations we will restrict to the original formulation (1.1), so that we can avail ourselves of an already well-established efficient (in terms of computational time) numerical scheme (Gerisch 2010). Here we use $c_{L}$ and $v_{L}$ to denote solutions to the local formulation and $c_{Ar}$ and $v_{Ar}$ to denote solutions to the nonlocal model with sensing radius *r*. We remark that a large number of related local and nonlocal models have been numerically studied to describe the invasion-type process considered here (e.g. Perumpanani et al. 1996; Anderson et al. 2000; Gerisch and Chaplain 2008; Painter et al. 2010): here the specific focus is to explore the convergence of nonlocal to local form as $r\rightarrow 0$, which, as far as we are aware, has not been systematically investigated.

As in the first test we use the initial values (6.1) and (6.2), choosing $x_c=L/2$, $\alpha =10$ in the latter, and consider the coefficients and functions as proposed in Example 5.5. Under these choices the resultant nonlinear diffusion coefficient for the *c*-equation in the classical local formulation (compare (5.2a)) becomes

6.3 $$\begin{aligned} {\tilde{D}}_c(c,v)= \frac{a^2(1+c)^2(1+c+v)^2-bc(1+cv)(S_{cc}+(S_{cc}-S_{cv})v)}{(1+cv)^2(1+c+v)^2}. \end{aligned}$$

Notably, this potentially becomes negative under an injudicious combination of adhesive strengths $S_{cc}$, $S_{cv}$, and of *a*, *b*. Likewise, the actual haptotaxis sensitivity function takes the form

6.4 $$\begin{aligned} {{\tilde{\chi }}}(c,v)= b\frac{S_{cv}+(S_{cv}-S_{cc})c}{(1+cv)(1+c+v)^2}. \end{aligned}$$

Again, depending on the relationship between $S_{cc}$ and $S_{cv}$, this can become negative, which would lead to repellent haptotaxis: cells effectively moving away from regions with large ECM gradients, a rather unexpected behaviour. This suggests that cell–tissue adhesions should dominate over cell–cell adhesions,^4^ as ’usual’ haptotaxis, i.e. towards the increasing tissue gradient, is known to be an essential component of cell migration, this applying to several types of cells moving through the ECM (tumor cells, mesenchymal stem cells, fibroblasts, endothelial cells, etc.) see e.g. Lamalice et al. (2007), Pickup et al. (2014) and Wen et al. (2015) and references therein.

**Fig. 3:**
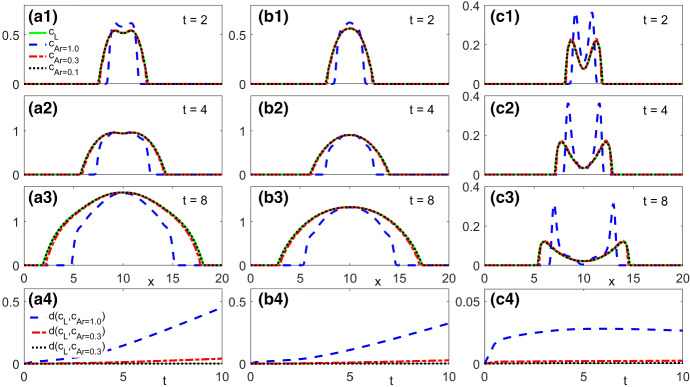
Convergence between nonlocal and local/classical formulations under negligible cell–cell adhesion, $S_{cc} = 0$, $S_{cv} = 10$. Functional forms as proposed in Example 5.5, with modifications specified in the subfigures. **a** Solutions for $r = 0.1, 0.3, 1.0$ at **a1** $t=2$, **a2** $t=4$ and **a3** $t=8$; **a4** distance between local/nonlocal solutions as a function of time. For these simulations, we take $a = 0.01$, $b = 1$, $\mu _c = 0.01$, $K_c = 2$, $\eta _c = 1$, $\mu _v = 0$, $\lambda _v = 1$. **b** Solutions for $r = 0.1, 0.3, 1.0$ at **b1** $t=2$, **b2** $t=4$ and **b3** $t=8$; (b4) Distance between local/nonlocal solutions as a function of time. Parameters as in **a** except $\mu _v = 1$, $K_v = 1$. **c** Solutions for $f_c = 0$ and $f_v(c,v)= - c v$, with the other parameters as in (**a**)

**Fig. 4:**
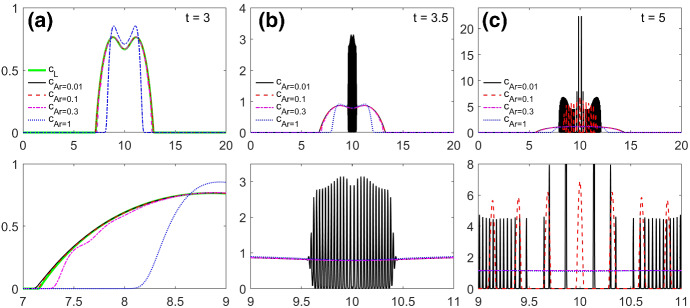
Time restricted convergence under moderate cell–cell adhesion, $S_{cc} = 2.5$, $S_{cv} = 10$. Top row shows solutions across the full spatial region ([0, 20]), the bottom row magnifies a relevant portion for clarity. Solutions to local and nonlocal models under the functional forms proposed in Example 5.5 for $r = 0.01, 0.1, 0.3, 1.0$ at **a** $t=3$, **b** $t=3.5$ and **c** $t=5$. In (**a**) solutions to the local model continue to exist and we observe convergence between local and nonlocal formulations. In (**b**, **c**) the solutions to the local model are noncomputable. Nonlocal models, however, can destabilise into a pattern of aggregates. Parameters: $a = 0.01$, $b = 1$, $\mu _c = 0.01$, $K_c = 2$, $\eta _c = 1$, $\mu _v = 0$, $\lambda _v = 1$ and adhesion parameters as above

Simulations are plotted in Fig. 3 where we show cell densities for the local model ($c_L$) and nonlocal model under three sensing radii:

$$\begin{aligned} c_{Ar=0.1}, c_{Ar=0.3}, c_{Ar=1.0}. \end{aligned}$$

In this first set of simulations we assume negligible cell–cell adhesion ($S_{cc} = 0$), which automatically ensures positivity for the diffusion coefficient of the equivalent local model, ${\tilde{D}}_c(c,v)$. We note that matrix renewal is absent ($\mu _{v} = 0$) in the left-hand column and present ($\mu _{v} > 0$) in the central column. In the right-hand column we show the greater generality of the results under vastly simplified kinetics, specifically setting $f_c(c,v) = 0$ and $f_v(c,v) = - c v$ (with the other functional forms as in Example 5.5). Simulations highlight the convergence between local and nonlocal models as $r\rightarrow 0$: for $r = 0.1$, the solution differences become negligible. However, distinctions emerge for large *r*, where we can expect significant discrepancy between the solutions. This suggests that the local model fails to accurately predict the behaviour in cases where cells sample over relatively large regions of their local environment.

Next, we extend to include a degree of cell–cell adhesion, setting functions and parameters as in Fig. 3, except now $S_{cc} > 0$. Notably this raises the possibility of a negative diffusion coefficient in the classical formulation and subsequent illposedness. Solutions under a representative set of parameters are shown in Fig. 4. For *t* below some critical time we observe convergence as before, with the nonlocal formulation converging to solutions of the local model as $r\rightarrow 0$. However, continued matrix degradation further depletes *v*, with the result that (6.3) can become negative. At this point (in this case $t\approx 3.2\ldots$) the local model becomes illposed and its solutions become incomputable (implying nonexistence of solutions). However, the nonlocal formulation appears to preserve wellposedness, consistent with previous theoretical studies where extending to a nonlocal formulation regularises a singular local model (e.g. Hillen et al. 2007). Solutions to the nonlocal model instead destabilise into a quasi-periodic pattern of cell aggregations, maintained through the cell–cell adhesion, and with a wavelength shrinking as $r \rightarrow 0$.

Finally, we remark that convergence of solutions extends beyond the specific functional forms and, as a representative example, we consider a minimalist setting based on linear/constant forms. Specifically, we set $D_c = a$ (constant), $\chi = 1$, $f_c = 0$, $g(c,v) = S_{cc} c + S_{cv} v$ and $f_v(c,v) = -\mu c v$. In this scenario, the diffusion and haptotaxis coefficients for the classical local formulation (5.2) reduce to

6.5 $$\begin{aligned} {\tilde{D}}_c(c,v)=a - S_{cc} c \quad \text{ and } \quad {\tilde{\chi }}(c,v)= S_{cv}. \end{aligned}$$

**Fig. 5:**
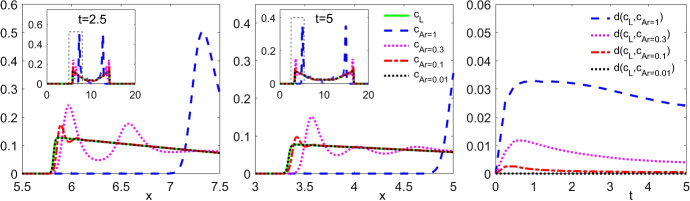
Convergence between nonlocal and local/classical formulations under a set of minimalistic linear functional forms ($D_c = 0.01,\ \chi = 1,\ f_c = 0,\ g(c,v) = S_{cc} c + S_{cv} v,\ f_v(c,v) = -\mu c v$). Negligible cell–cell adhesion, $S_{cc}=0,\ S_{cv}=10$: solutions shown at (left) $t=2.5$ and (middle) $t=5$, with the distance between solutions to the nonlocal and local model shown in the right panel

Positivity is only guaranteed under appropriate parameter selection. Such a case is illustrated in Fig. 5 where we assume negligible cell–cell adhesion ($S_{cc} = 0$). Clearly, we observe convergence between the nonlocal and local formulations as $r\rightarrow 0$. Inappropriate parameter selection, however, generates backward diffusion in the local model and solutions are consequently incomputable. In all cases considered in this test the cells do not reach the boundary region where the difference between the nonlocal formulations (1.1) and (5.1) can play a role. Thus, we expect the same solution if reformulation (5.1) is applied instead.

## Discussion

In this work we provide a rigorous limit procedure which links nonlocal models involving adhesion or a nonlocal form of chemotaxis gradient to their local counterparts featuring haptotaxis, respectively chemotaxis in the usual sense. As such, our paper closes a gap in the existing literature. Moreover, it offers a unified treatment of the two types of models and extends the previous mathematical framework to settings allowing for more general, solution dependent, coefficient functions (diffusion, tactic sensitivity, adhesion velocity, nonlocal taxis gradient, etc.). Finally, we provide simulations illustrating some of our theoretical findings in 1D.

Our reformulations in terms of ${\mathcal {T}}_r$ and ${\mathcal {S}}_r$ reveal the tight relationship between the nonlocal operators ${\mathcal {A}}_r$ and $\mathring{\nabla }_{r}$ and the (local) gradient. This suggests that both nonlocal descriptions (adhesion, chemotaxis) actually encompass the dependence on the signal gradients rather than on the signal concentration/density itself, which is in line with the biological phenomenon. Indeed, through their transmembrane elements (e.g. receptors, ion channels etc.) the cells are mainly able to perceive and respond to differences in the signal at various locations or within more or less confined areas rather than measure effective signal concentrations. Along with the mentioned solution dependency of the nonlocal model coefficients, the influence of the gradient possibly reflects into contributions of the adhesion/nonlocal chemotaxis to the (nonlinear) diffusion in the local setting obtained through the limiting procedure.

The set $\varOmega _r$ (as introduced in Sect. 2) can be regarded as the ’domain of restricted sensing’, meaning that there cells a priori sense only what happens inside $\varOmega$, the domain of interest. The measure of this subdomain is a decreasing function of the sensing radius *r*. When $r\rightarrow 0$ the set $\varOmega _r$ tends to cover the whole domain $\varOmega$, whereas as *r* increases the cells can sense at increasingly larger distances; correspondingly, $\varOmega _r$ shrinks. For $r>{\text {diam}}(\varOmega )$ the restricted sensing domain is empty: everywhere in $\varOmega$ the cells can perceive signals not only from any point within $\varOmega$ but potentially also from the outside. In this paper, however, we look at models with no-flux boundary conditions. This corresponds, e.g., to the impenetrability of the walls of a Petri dish or that of comparatively hard barriers limiting the areas populated by migrating cells, e.g. bones or cartilage material. As a result, the cells in the boundary layer $\varOmega {\setminus } \varOmega _r$ have a much reduced ability to stretch their protrusions outside $\varOmega$ and thus gain little information from without. To simplify matters, we assume in this work that there is no such information or it is insufficient to trigger any change in their behaviour. In the definitions of ${\mathcal {T}}_r$ and ${\mathcal {S}}_r$ this corresponds to the integrands being set to zero in $\varOmega \backslash {\overline{\varOmega }}_r$.

It is important to note that for points $x\in \varOmega \backslash {\overline{\varOmega }}_r$ the influence of a signal *p* in a direction $y\in S_1$ is not taken into account by $\mathring{\nabla }_{r}$ at all if $x+ry\not \in {\overline{\varOmega }}$. If ${\mathcal {S}}_r$ is used instead, then its contribution to the average is given by

$$\begin{aligned} \tilde{y}:=n \left( \int _0^1\chi _{\varOmega }\nabla p(x+rsy)\,ds\cdot y\right) y. \end{aligned}$$

Thus, thanks to integration w.r.t. *s*, the resulting vector $\tilde{y}$ assembles the impact of those parts of the segment connecting *x* and $x+ry$ which are contained in $\varOmega$. It is parallel to *y*, and it may have the same or the opposite orientation. In particular this means that although for a certain range of directions large parts of the sensing region of a cell are actually outside $\varOmega$, this may still strongly influence the speed and actual direction of the drift. The effect of integration w.r.t. *s* in ${\mathcal {T}}_r$ is less obvious, since in this case the average w.r.t. *y* is computed over the ball $B_1$. This already achieves the covering of the whole sensing region by allowing a cell to gather information about the signal not only in any direction *y*/|*y*|, but also at any distance less than *r*. The additional integration over the path $x+rsy$, $s\in [0,1]$, appears to mean that cells at $x\in \varOmega _r$ are able to measure the average of the signal gradient all along such line segment rather than its value directly at the ending point. Indeed, from a biological viewpoint this description seems to make more sense, as cells do not jump from one position to another, nor do they send out their protrusions in a discontinuous way bypassing certain space points along a chosen direction. Averages over cell paths are then averaged w.r.t. *y*, which finally determines the direction of population movement. Example 3.4 indicates that the effect of even an extremely concentrated signal gradient is mollified by averaging. This agrees with our expectations from using non-locality. In higher dimensions $n\ge 2$, the two-stage averaging in ${{{\mathcal {T}}}}_r$ (w.r.t. *s* and *y*) produces a direction field which is smooth away from the concentration point and also weakens but still keeps the singularity there. In contrast, averaging only w.r.t. *y* leads instead to jump discontinuities at a unit distance from the accumulation point. Moreover, we remark that without integrating w.r.t. *s* in ${{{\mathcal {T}}}}_r(\nabla \cdot )$ one cannot regain ${{{\mathcal {A}}}}_r$.

The effect observed in Example 3.3 further supports the conjecture that the nonlocal operators which act directly on the signal gradients might actually be a more appropriate modelling tool. While inside the subdomain $\varOmega _r$ there is no difference (recall Lemmas 3.1 and 3.2), inside the boundary layer $\varOmega \backslash {\overline{\varOmega }}_r$ the limiting behaviour as $r\rightarrow 0$ is qualitatively distinct. Indeed, Example 3.3 shows that using, e.g., ${\mathcal {A}}_r$, leads, for $r\rightarrow 0$, to unnatural sharp singularities at the boundary of $\varOmega$ even in the absence of signal gradients, whereas this does not happen if ${\mathcal {T}}_r$ is used instead. Simulations in Sect. 6.1 (see Fig. 2) confirm our theoretical findings and show a substantial difference between the solutions obtained with the two nonlocal formulations involving (1.2) and (3.2), respectively. The choice (3.2) is motivated above all from a mathematical viewpoint (as it enables a rigorous, well-justified passage to the limit for $r\rightarrow 0$), but it also seems to make sense biologically, as our above comments and the simulations performed for the particular setting in Sect. 6.1 suggest.

In this work we have only dealt with models that include a nonlocality in the chemotaxis or cell–cell and/or cell–tissue adhesion terms and assumed the diffusion to be local. This is in line with most of the previously developed nonlocal models for cell migration, albeit they usually cover just linear diffusion. If cell–cell adhesion is present, this means that the cell flux contains the local cell gradient, as well as some averaging of it. The latter is described in our case by a suitably chosen operator ${\mathcal {T}}_r$. A possible model extension could involve a diffusion flux which is also nonlocal and has a similar form. This would mean that the cell flux is completely devoid of the local gradient. From the modelling point of view this could be seen as a population pressure acting^5^ in a nonlocal manner: each cell is sensing the population mass not only at its current position, but over a whole region (of radius *r*) around that location. This is actually true in vivo, where cells sample their biological environment by extending protrusions as far as several cell lengths. While cell–cell adhesions certainly play a role in this process and contribute to self-diffusion (as in the example handled in Sect. 6.2), there might be yet other ways of interaction by which the cells are able to perceive smaller or larger aggregates of their own kind. In this context one could think about replacing the local gradient by a nonlocal operator, e.g. of the form ${\mathcal {T}}_r(\nabla )$. However, the analysis of such a model would be considerably more involved and it is to expect that existence of solutions can be established only under rather restrictive assumptions.

## Footnotes

1 In Sect. 3 we will see that this is, indeed, the ’right’ normalisation. If we assume, as in Armstrong et al. (2006), that this function is a constant involving some viscosity related proportionality, then this choice provides the value of that constant.

2 As usual, here and below the expression $\frac{s+1}{s}$ means infinity if $s=0$.

3 Unless environmental influences dictate conversion to a collective type of motion.

4 An analogous behaviour was suggested by the two-scale structured population model with adhesion introduced in Engwer et al. (2017).

5 Unlike Fick’s classical law which typically connects the flux over the domain boundary with the diffusion inside the domain.
